# TUFT1 regulates cancer progression by suppressing centrosome amplification and mitotic spindle multipolarity

**DOI:** 10.1038/s41419-025-08010-3

**Published:** 2025-09-29

**Authors:** Shaojie Feng, Mengmeng Zhao, Donghui Zhang, Yan Zhang, Lingyuan Min, Xianqiang Liu, Huan Shi, Tianning Wang

**Affiliations:** 1 Breast Disease Diagnosis and Treatment Center/Department of Thyroid Surgery, Central Hospital Affiliated to Shandong First Medical University, Jinan, China; 2 Research Center of Translational Medicine, Central Hospital Affiliated to Shandong First Medical University, Jinan, China; 3 Zhanjiang Institute of Clinical Medicine, Central People’s Hospital of Zhanjiang, Guangdong Medical University Zhanjiang Central Hospital, Zhanjiang, China; 4 Department of Internal Medicine-Oncology, Shandong Cancer Hospital and Institute, Shandong First Medical University and Shandong Academy of Medical Sciences, Jinan, China

**Keywords:** Centrosome, Mitotic spindle, Phosphorylation, Oncogenes

## Abstract

Centrosome amplification (CA) has been observed in various solid tumors and contributes to chromosomal instability (CIN) and poor clinical prognosis in patients with cancer. CA also inhibits cell proliferation by inducing cell-cycle arrest and cell death. However, the mechanism of regulation of centrosome number in cancer cells and its effect on CIN and cell proliferation remains elusive. Here, we report that tuftelin (TUFT1) is a novel centrosomal protein that localizes to the proximal ends of parent centrioles. TUFT1 prevents CA and mitotic spindle multipolarity by suppressing premature polo-like kinase 1 activation. In addition, TUFT1 is phosphorylated by NIMA-related kinase 2 (NEK2), and the phosphorylation status of TUFT1 is essential for coordinating centrosome number and cell proliferation in cervical and breast cancers. Data from clinical breast cancer samples indicate that the combined detection of TUFT1 and NEK2 expression reflects tumor malignancy and patient survival with higher precision. Overall, these results reveal a crucial role of TUFT1 in the regulation of tumor progression through centrosome number control. Thus, TUFT1 represents a promising target for diagnostic and therapeutic approaches for cancers.

## Introduction

The centrosome, consisting of a pair of centrioles embedded in the pericentriolar material, is the main microtubule organizing center in animal cells [1]. The centrosome plays a crucial role in the regulation of various cellular processes, such as cell division, cell motility, and ciliogenesis [2–4]. Centrosome number is under strict spatiotemporal control during each cell cycle, and centrosome amplification (CA) is one of the main causes of mitotic spindle multipolarity and chromosomal instability (CIN) in cancer cells [5]. CIN contributes to intratumoral heterogeneity and drives phenotypic adaptations, leading to treatment failure and disease recurrence [6]. However, CA also induces an anti-proliferative response through PIDDosome/p53 pathway-dependent cell-cycle arrest and cell death [7]. In fact, CA has been observed in various cancers and is closely associated with progression and clinical prognosis in patients [8–11]. The mechanism of regulation of centrosome number in cancer cell populations and its resulting effects on the balance between CIN and cell proliferation remains elusive.

Several mechanisms have been reported to contribute to CA, including dysregulation of centrosome duplication-associated proteins (such as polo-like kinase 4 [PLK4] and spindle assembly abnormal protein 6 [SAS-6]), perturbation of cell-cycle progression, and mitotic defects [12–14]. In addition, premature activation of PLK1 also leads to CA. PLK1, a master kinase activated in late G2 phase and mitosis, coordinates centrosome maturation, spindle assembly, and cytokinesis [15–17]. However, premature PLK1 activation in early cell-cycle phases induces premature centriole disengagement and centriole reduplication [18–21]. PLK1 is overexpressed in various cancers, and growing evidence supports coupling of PLK1 to tumorigenesis, tumor progression, and chemoresistance [22–27]. Therefore, investigating the regulation of PLK1 expression/activity in cancers is of vital importance.

Tuftelin (TUFT1) was initially characterized as an acidic protein involved in the development and mineralization of tooth tissues [28, 29]. In addition, TUFT1 is also expressed in non-mineralized normal and cancerous tissues [30, 31]. TUFT1 is highly expressed in several cancers, such as thyroid, hepatocellular, pancreatic, breast, and stomach carcinomas and predicts worse clinical status and poor prognosis [32–35]. TUFT1 exerts oncogenic roles by activating PI3K/AKT, HIF-1/Snail, and Rab5/Rac1/β-catenin pathways or by upregulating the expression of the lncRNA DANCR [32–40]. Besides, TUFT1 promotes hepatocellular carcinoma progression through regulation of lipogenesis and focal adhesion maturation [41]. Therefore, TUFT1 is a potential target for diagnostic and therapeutic approaches for cancers. However, its interactome and biological functions have not yet been fully characterized.

Here, we report that TUFT1 is a novel centrosomal protein that localizes to the proximal ends of parent centrioles. TUFT1 prevents CA and mitotic spindle multipolarity by suppressing premature PLK1 activation. In addition, TUFT1 is phosphorylated by NIMA-related kinase 2 (NEK2), and the phosphorylation status of TUFT1 is essential for coordinating centrosome number and cell proliferation in cervical and breast cancers. Hyperphosphorylation of TUFT1 favors the occurrence of CA and inhibits cell proliferation, whereas hypophosphorylation of TUFT1 has the opposite effect. Upregulation of the NEK2/TUFT1 axis in breast cancer is associated with poor outcomes, implying that for the cancer cell population the disadvantage conferred by TUFT1 hyperphosphorylation on cell proliferation may be overcome by its advantage of increasing CA and CIN.

## Results

### TUFT1 is recruited to the proximal ends of parent centrioles by C-Nap1

**Fig. 1:**
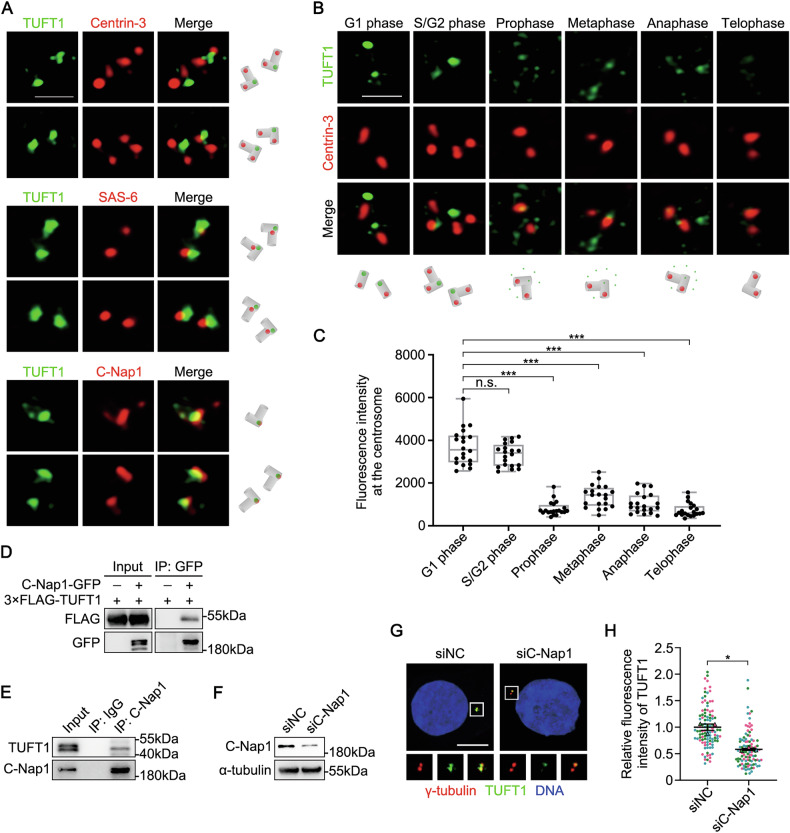
TUFT1 is recruited to the proximal ends of parent centrioles by C-Nap1. **A** Immunostaining of TUFT1 (green) and Centrin-3, SAS-6, or C-Nap1 (red) in HeLa cells. Scale bar, 1 μm. **B** Immunostaining of TUFT1 (green) and Centrin-3 (red) in HeLa cells throughout the cell cycle. Scale bar, 1 μm. **C** Quantification of the fluorescence intensity of TUFT1 in (**B**) (*n* = 20 cells). Data are shown as the mean (min to max). **D** Lysates of HEK293T cells overexpressing C-Nap1-GFP and 3×FLAG-TUFT1 were subjected to immunoprecipitation (IP) and immunoblotting, as indicated. **E** Lysates of HeLa cells were subjected to IP and immunoblotting, as indicated. **F** Immunoblots of C-Nap1 in HeLa cells transfected with negative control (NC)- or C-Nap1-siRNA. α-tubulin served as a loading control. **G** Immunostaining of TUFT1 (green) and γ-tubulin (red) in HeLa cells transfected with NC- or C-Nap1-siRNA. DNA was stained with 4′,6-diamidino-2-phenylindole (DAPI) (blue). Scale bar, 10 μm. **H** Quantification of the fluorescence intensity of TUFT1 in (**G**) (*n* > 100 cells from three independent experiments). Data are shown as the mean ± s.e.m. *P*-values are shown in the figure (one-way ANOVA in (**C**); paired two-tailed Student’s *t*-test in (**H**)).

Previous studies have characterized TUFT1 as a cytoplasmic and secretory protein in normal and cancerous soft tissues [30, 31]. However, we also observed clear signals corresponding to TUFT1 expression on the centrosome of cultured cells following fixation and permeabilization in cold methanol (Fig. S1A). Next, we characterized the distribution of TUFT1 on the centrosome using 3D structured illumination microscopy (3D-SIM). TUFT1 mainly appeared in the form of dot-like structures, which co-localized well with the centriole proximal-end marker C-Nap1 but exhibited almost no overlap with the centriole distal-end marker Centrin-3 (Fig. 1A). Additionally, TUFT1 partially co-localized with SAS-6, which is located outside the proximal regions of the parent centrioles (Fig. 1A). These results indicate that TUFT1 localizes to the proximal ends of parent centrioles.

Furthermore, we observed centrosomal localization of TUFT1 throughout the cell cycle. The signal intensity of TUFT1 on the centrosome remained constant during interphase but decreased significantly upon entry into mitosis (Fig. 1B, C). Next, we investigated the region of TUFT1 responsible for centrosomal targeting. TUFT1 contains 390 amino acids (aa) with two coiled-coil domains. We constructed two truncated mutants based on the distribution of the coiled-coil domains and analyzed their subcellular localization. The results revealed that TUFT1 localizes to the centrosome through its N-terminus (1–150 aa) (Fig. S1B).

Given that TUFT1 co-localized with C-Nap1 at the proximal ends of parent centrioles, we investigated the recruitment relationship between the two proteins. We first confirmed the biochemical association between TUFT1 and C-Nap1 using exogenous and endogenous co-immunoprecipitation assays (Fig. 1D, E). C-Nap1 depletion significantly decreased the centrosomal localization of TUFT1 (Fig. 1F–H), indicating that C-Nap1 functions upstream of TUFT1. Next, we mapped the regions of TUFT1 that are involved in its interaction with C-Nap1. We found that TUFT1 co-immunoprecipitated with C-Nap1 via its N-terminus, the region that is also involved in centrosomal targeting (Fig. S1B and S1C). These results further confirmed that TUFT1 is recruited by C-Nap1 to the centrosome.

### TUFT1 suppresses CA and mitotic spindle multipolarity

**Fig. 2:**
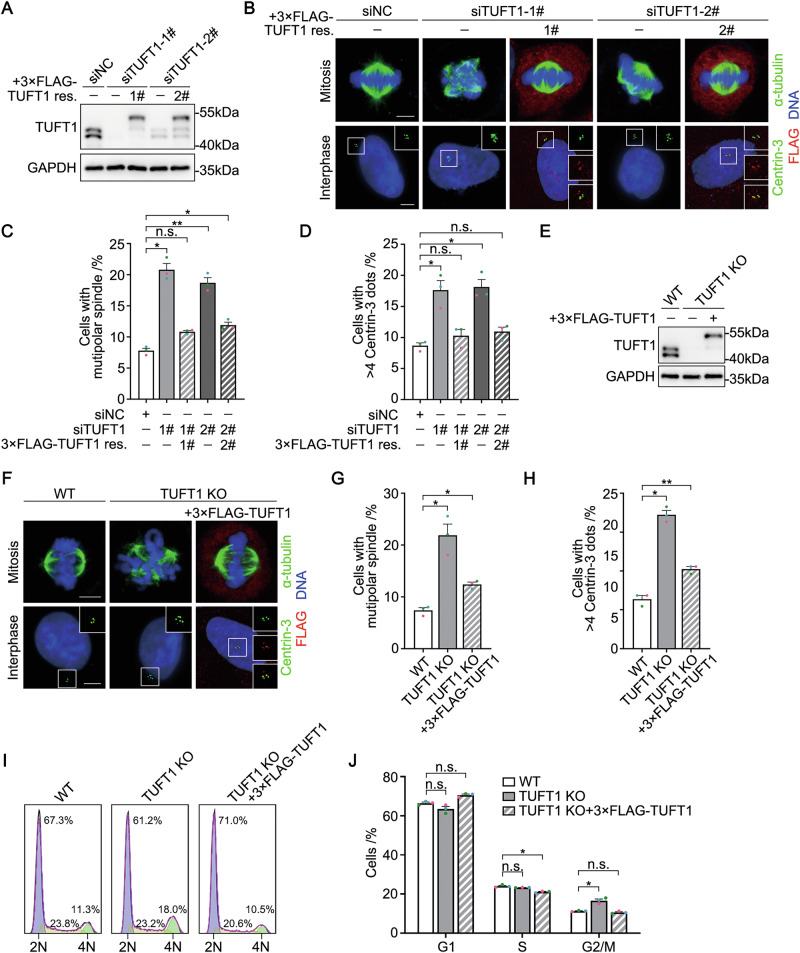
Loss of TUFT1 induces centrosome amplification and mitotic spindle multipolarity. **A** Immunoblots of TUFT1 in HeLa cells transfected with negative control (NC)- or TUFT1-siRNA. SiRNA-resistant 3×FLAG-TUFT1 was transiently transfected into knockdown cells to rescue the phenotype. GAPDH served as a loading control. **B** Immunostaining of α-tubulin (green, upper panel) or Centrin-3 (green, bottom panel) in HeLa cells transfected with NC- or TUFT1-siRNA. SiRNA-resistant 3×FLAG-TUFT1 (red) was transiently transfected into knockdown cells to rescue the phenotype. DNA was stained with DAPI (blue). Scale bar, 5 μm. **C** Quantification of cells with multipolar spindle in (**B**) (*n* > 300 cells from three independent experiments). **D** Quantification of Centrin-3 foci number in (**B**) (*n* > 300 cells from three independent experiments). **E** Immunoblots of TUFT1 in wild-type (WT) and TUFT1-knockout (KO) HeLa cells. GAPDH served as a loading control. 3×FLAG-TUFT1 was transiently transfected into KO cells to rescue the phenotype. **F** Immunostaining of α-tubulin (green, upper panel) or Centrin-3 (green, bottom panel) in WT or TUFT1-KO HeLa cells. 3×FLAG-TUFT1 (red) was transiently transfected into KO cells to rescue the phenotype. DNA was stained with DAPI (blue). Scale bar, 5 μm. **G** Quantification of cells with multipolar spindle in (**F**) (*n* > 300 cells from three independent experiments). **H** Quantification of Centrin-3 foci number in (**F**) (*n* > 300 cells from three independent experiments). **I** Flow cytometric analysis of WT and TUFT1-KO HeLa cells. 3×FLAG-TUFT1 was transiently transfected into KO cells to rescue the phenotype. **J** Quantification of cell cycle in (**I**) (three independent experiments). Data in (**C**), (**D**), (**G**), (**H**), and (**J**) are shown as the mean ± s.e.m. *P*-values are shown in the figure (one-way ANOVA).

To investigate the function of TUFT1 on the centrosome, we depleted endogenous TUFT1 in HeLa cells using short interfering RNAs (siRNAs) (Fig. 2A) and investigated whether TUFT1 regulates centrosome number and mitotic spindle assembly. The proportion of mitotic cells with multipolar spindles increased from ~8% to 20% following TUFT1 knockdown (Fig. 2B, C). The proportion of interphase cells with more than four Centrin-3 foci increased from ~9% to 18% following TUFT1 knockdown (Fig. 2B, D), indicating that TUFT1 negatively regulates centriole number. Next, we explored whether CA occurs after TUFT1 depletion. We quantified the foci numbers of PLK4 and SAS-6, both of which are critical for centriole duplication initiation. In wild-type cells, the two proteins consistently localize as single dot at each parent centriole, maintaining strict control over procentriole number (Fig. S1D and S1E). Strikingly, TUFT1 depletion induced ectopic accumulation of both PLK4 and SAS-6, manifested as multiple distinct foci surrounding parent centrioles (Fig. S1D and S1E), indicating multiple procentriole formation events within a single cell cycle. All the phenotypes were rescued by transient transfection with siRNA-resistant TUFT1 (Figs. 2A–D, S1D, and S1E).

To further confirm the effect of TUFT1 on centrosome number and mitotic spindle morphology, we generated TUFT1-knockout HeLa cells using the CRISPR/Cas9 approach and validated the knockout efficiency by sequencing, immunostaining, and immunoblotting (Figs. 2E and S2A–S2C). Similar phenotypes were observed in TUFT1-knockout HeLa cells, which were rescued by the reintroduction of exogenous TUFT1 (Figs. 2F–H, S2D, and S2E). These results indicate that the loss of TUFT1 induces CA, which further leads to multipolar mitosis. Subsequent flow cytometric analysis revealed no significant change in the proportion of S-phase cells following TUFT1 knockout (Fig. 2I, J), indicating that TUFT1 knockout-induced CA was not a result of S-phase arrest. Additionally, TUFT1 knockout did not affect the centrosomal localization of C-Nap1 (Fig. S2F and S2G), further confirming that TUFT1 acts downstream of C-Nap1.

### TUFT1 interacts with PLK1

**Fig. 3:**
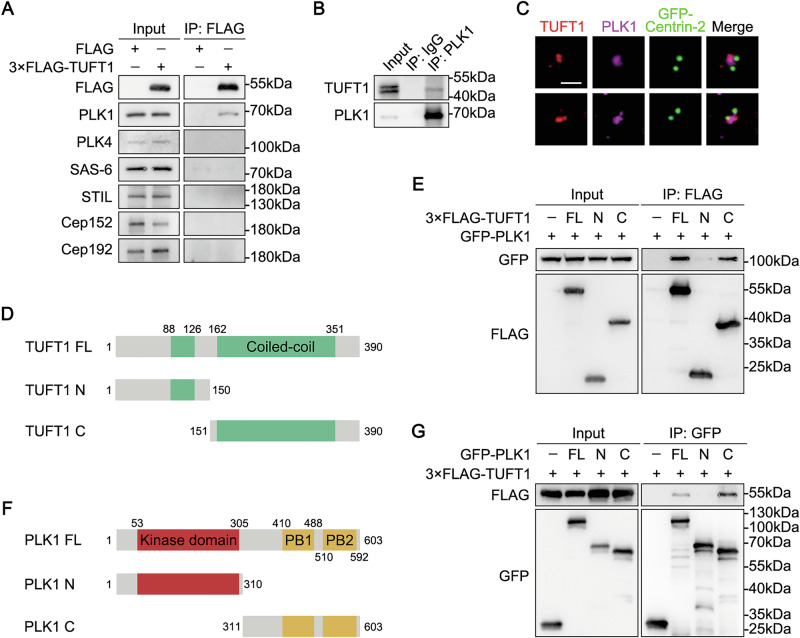
TUFT1 interacts with PLK1. **A** Lysates of HEK293T cells overexpressing 3×FLAG-TUFT1 were subjected to immunoprecipitation (IP) and immunoblotting, as indicated. **B** Lysates of HeLa cells were subjected to IP and immunoblotting, as indicated. **C** Immunostaining of TUFT1 (red) and PLK1 (purple) in HeLa cells overexpressing GFP-Centrin-2 (green). Scale bar, 1 μm. **D** Schematic of TUFT1 and its truncated mutants (full-length, FL; N-terminus, N; C-terminus, C). **E** Lysates of HEK293T cells overexpressing GFP-PLK1 and 3×FLAG-TUFT1 (FL, N, or C) were subjected to IP and immunoblotting, as indicated. **F** Schematic of PLK1 and its truncated mutants (FL, N and C). PB, POLO box. **G** Lysates of HEK293T cells overexpressing 3×FLAG-TUFT1 and GFP-PLK1 (FL, N, or C) were subjected to IP and immunoblotting, as indicated.

Having confirmed the role of TUFT1 in the regulation of centrosome number, we performed co-immunoprecipitation assays to investigate potential associations between TUFT1 and several key centrosome duplication regulators (Cep152, Cep192, PLK1, PLK4, SAS-6, and STIL). The results showed that exogenous TUFT1 was selectively co-immunoprecipitated with PLK1 but not the other proteins (Fig. 3A). PLK1 is a master serine/threonine-protein kinase that regulates centrosome maturation and mitotic spindle assembly [15–17]. Subsequently, an interaction between endogenous TUFT1 and PLK1 was also detected (Fig. 3B). Furthermore, we found that TUFT1 co-localized well with PLK1 on the centrosome (Fig. 3C).

Next, we mapped the regions of TUFT1 and PLK1 that are involved in their interaction. TUFT1 interacted with PLK1 through its C-terminus (151–390 aa) (Fig. 3D, E), and PLK1 bound to TUFT1 through its C-terminus (311–603 aa) (Fig. 3F, G). Thus, the N-terminus of TUFT1 mediates centrosomal localization (Fig. S1B), while its C-terminus interacts with PLK1 (Fig. 3D, E). Consistent with these findings, neither N-terminal nor C-terminal truncation mutants of TUFT1 rescued the phenotypes caused by TUFT1 knockout (Fig. S2H–S2K). Fusion of the TUFT1 C-terminus with the conserved centrosomal targeting motif (the PACT domain) [42] (PACT-C) restored centrosome number and spindle morphology (Fig. S2H–S2K), suggesting that both centrosomal localization and interaction with PLK1 are indispensable for the functional integrity of TUFT1.

### TUFT1 suppresses premature activation of PLK1

**Fig. 4:**
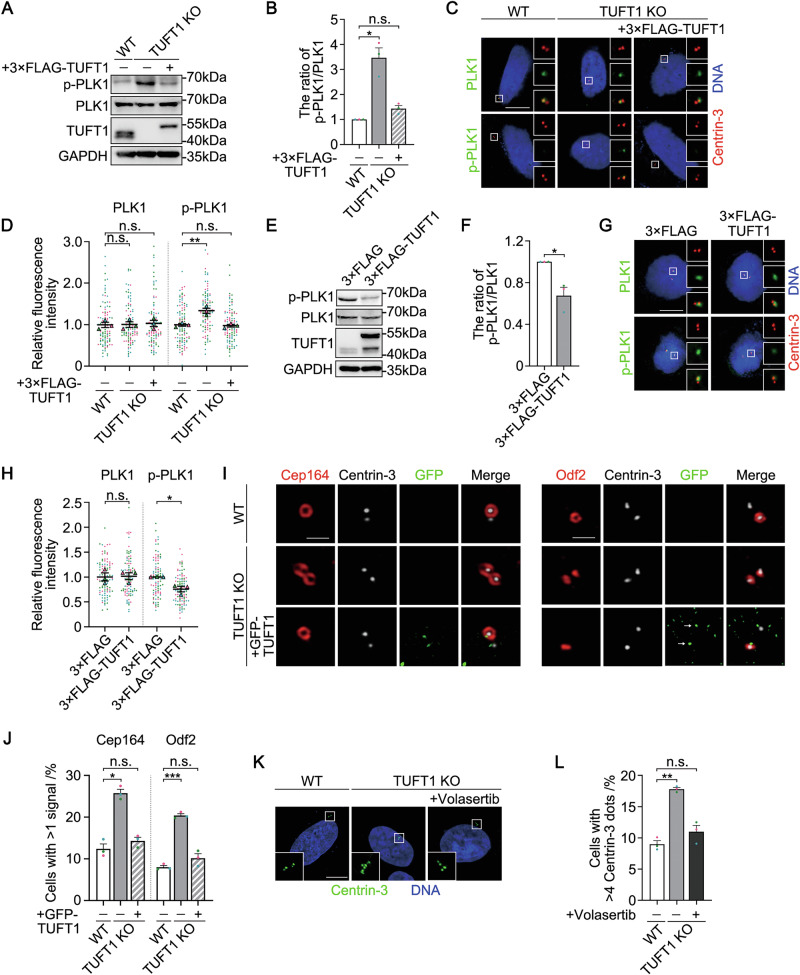
TUFT1 suppresses premature activation of PLK1. **A** Evaluation of the effects of TUFT1 knockout (KO) on PLK1 activation in HeLa cells by immunoblotting. Phospho-PLK1 (p-PLK1) indicates PLK1 activation. 3×FLAG-TUFT1 was transiently transfected into KO cells to rescue the phenotype. GAPDH served as a loading control. **B** Quantification of the ratio of p-PLK1/PLK1 in (**A**) (three independent experiments). **C** Immunostaining of PLK1 (green, upper panel) or p-PLK1 (green, bottom panel) and Centrin-3 (red) in wild-type (WT) and TUFT1-KO HeLa cells. 3×FLAG-TUFT1 was transiently transfected into KO cells to rescue the phenotype. DNA was stained with DAPI (blue). Scale bar, 10 μm. **D** Quantification of the fluorescence intensities of PLK1 and p-PLK1 in (**C**) (*n* > 100 cells from three independent experiments). **E** Evaluation of the effects of TUFT1 overexpression on PLK1 activation in HeLa cells by immunoblotting. GAPDH served as a loading control. **F** Quantification of the ratio of p-PLK1/PLK1 in (**E**) (three independent experiments). **G** Immunostaining of PLK1 (green, upper panel) or p-PLK1 (green, bottom panel) and Centrin-3 (red) in HeLa cells overexpressing 3×FLAG-TUFT1. DNA was stained with DAPI (blue). Scale bar, 10 μm. **H** Quantification of the fluorescence intensities of PLK1 and p-PLK1 in (**G**) (*n* > 100 cells from three independent experiments). **I** Immunostaining of Cep164 (red, left panel), Odf2 (red, right panel), and Centrin-3 (white) in WT and TUFT1-KO HeLa cells. GFP-TUFT1 (green) was transiently transfected into KO cells to rescue the phenotype. Arrows: centrosome-localized exogenous TUFT1. Scale bar: 1 μm. **J** Quantification of Cep164 and Odf2 signal numbers in (**I**) (*n* > 300 cells from three individual experiments). **K** Immunostaining of Centrin-3 (green) in WT and TUFT1-KO HeLa cells treated with or without volasertib. DNA was stained with DAPI (blue). Scale bar, 10 μm. **L** Quantification of Centrin-3 foci number in (**K**) (*n* > 300 cells from three independent experiments). Data in (**B**), (**D**), (**F**), (**H**), (**J**) and (**L**) are shown as the mean ± s.e.m. *P*-values are shown in the figure (one-way ANOVA in (**B**), (**D**), (**J**) and (**L**); paired two-tailed Student’s *t*-test in (**F**) and (**H**)).

Given the observed interaction between TUFT1 and PLK1, we investigated whether TUFT1 affects centrosome number by regulating PLK1 activity. We found that phosphorylation of PLK1 at threonine residue 210 (T210) was upregulated following TUFT1 knockout or knockdown (Figs. 4A, B, S3A, and S3B), indicating the hyperactivation of PLK1. We also measured the amount of total and phosphorylated PLK1 on the centrosome. Although PLK1 localizes to the centrosome in G1 phase, phosphorylated PLK1 is barely detectable, as PLK1 is inactivated at this time point [15]. Loss of TUFT1 did not affect the centrosomal localization of PLK1; however, the amount of phosphorylated PLK1 on the centrosome was markedly enhanced (Figs. 4C, D, S3C, and S3D), indicating that TUFT1 depletion induces premature activation of PLK1. Conversely, TUFT1 overexpression suppressed PLK1 activity in prophase (Fig. 4E–H), further confirming that TUFT1 negatively regulates PLK1 activity.

It was reported that constitutive activation of PLK1 resulted in premature accumulation of distal and subdistal appendages to parent centrioles in cycling HeLa cells [21]. Consistent with these findings, the proportion of G1-phase cells harboring more than one Cep164 or Odf2 signal significantly increased following TUFT1 knockout or knockdown, suggesting premature PLK1 activation (Figs. 4I, J, S3E, and S3F).

Next, we investigated whether the inhibition of PLK1 rescues CA induced by TUFT1 depletion. The proportion of TUFT1-knockout cells with excess centrioles was reversed by treatment with the PLK1 inhibitor volasertib (from ~18% to 11%) (Fig. 4K, L). Volasertib treatment also suppressed CA induced by TUFT1 knockdown (from ~15% to 8.5%) (Fig. S3G and S3H). By contrast, inhibition of PLK1 did not alter the protein level or centrosome localization of TUFT1 (Fig. S3I–S3K), suggesting that TUFT1 acts upstream of PLK1. Taken together, these results suggest that TUFT1 suppresses CA by inhibiting premature activation of PLK1.

### TUFT1 is phosphorylated by NEK2

**Fig. 5:**
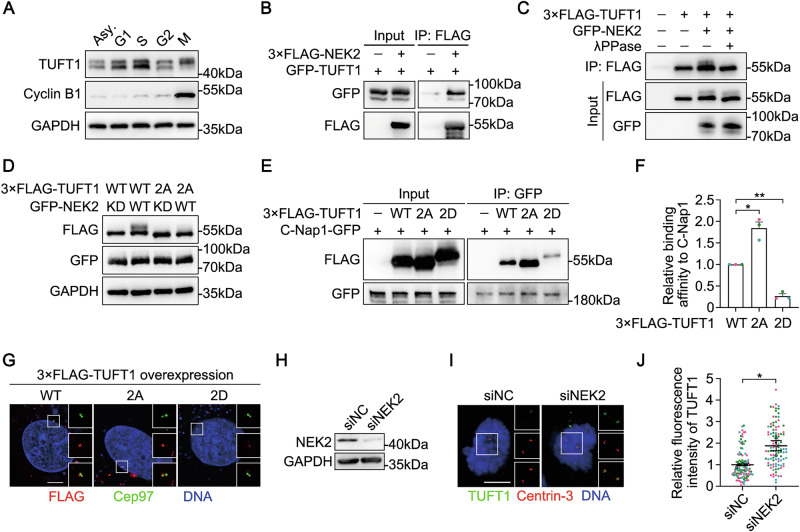
NEK2-mediated phosphorylation promotes the removal of TUFT1 from the centrosome. **A** Immunoblots of TUFT1 in synchronous HeLa cells. Cyclin B1 served as a marker for M phase. GAPDH served as a loading control. **B** Lysates of HEK293T cells overexpressing GFP-TUFT1 and 3×FLAG-NEK2 were subjected to immunoprecipitation (IP) and immunoblotting, as indicated. **C** HEK293T cells overexpressing 3×FLAG-TUFT1 and GFP-NEK2 were subjected to IP with anti-FLAG antibody, then treated with λPPase. Samples were immunoblotted with anti-FLAG and anti-GFP antibodies. **D** Lysates of HEK293T cells overexpressing 3×FLAG-TUFT1 (WT or 2A) and GFP-NEK2 (WT or the kinase-dead [KD] mutant) were immunoblotted with the indicated antibodies. GAPDH served as a loading control. **E** Lysates of HEK293T cells overexpressing C-Nap1-GFP and 3×FLAG-TUFT1 (WT, 2A, or 2D) were subjected to IP and immunoblotting, as indicated. **F** Quantification of the binding affinity to C-Nap1 in (**E**) (three independent experiments). **G** Immunostaining of Cep97 (green) in HeLa cells overexpressing 3×FLAG-TUFT1 (WT, 2A, or 2D) (red). DNA was stained with DAPI (blue). Scale bar, 5 μm. **H** Immunoblots of NEK2 in HeLa cells transfected with negative control (NC)- or NEK2-siRNA. GAPDH served as a loading control. **I** Immunostaining of TUFT1 (green) and Centrin-3 (red) in HeLa cells transfected with NC- or NEK2-siRNA. DNA was stained with DAPI (blue). Scale bar, 10 μm. **J** Quantification of the fluorescence intensity of TUFT1 in (**I**) (*n* > 100 cells from three independent experiments). Data in (**F**) and (**J**) are shown as the mean ± s.e.m. *P*-values are shown in the figure (one-way ANOVA in (**F**); paired two-tailed Student’s *t*-test in (**J**)).

Analysis of TUFT1 protein level in different cell-cycle phases revealed an up-shifted band in mitotic cells (Fig. 5A), implying that TUFT1 may be phosphorylated in mitosis. We performed co-immunoprecipitation assays to identify TUFT1-interacting centrosomal kinases and found that TUFT1 co-immunoprecipitated with NEK2 (Fig. 5B). To investigate whether TUFT1 is a substrate of NEK2, we co-overexpressed NEK2 and TUFT1. The results showed an up-shifted band corresponding to exogenous TUFT1 when co-overexpressed with NEK2, which was abrogated following λPPase treatment (Fig. 5C), suggesting that NEK2 phosphorylates TUFT1.

We compared the amino acid sequences of TUFT1 from different species for the NEK2 consensus phosphorylation motif [43], and identified five possible sites that may be phosphorylated by NEK2, including T29, S70, T175, T200, and S312 (Fig. S4A). We then generated TUFT1 phosphorylation-defective mutants by converting these sites to alanines and co-overexpressed them with NEK2. The phosphorylated band corresponding to TUFT1 was downregulated when either T29 or S70 was mutated, whereas it was completely eliminated when both sites were mutated (TUFT1-2A) (Fig. S4B). In addition, the up-shifted band corresponding to exogenous TUFT1 was detectable only when co-overexpressed with the wild-type, but not kinase-dead NEK2 (Fig. 5D), suggesting that these two sites in TUFT1 are specifically phosphorylated by NEK2.

As the centrosomal localization of TUFT1 was found to decrease upon entry into mitosis (Fig. 1B, C), and NEK2 exhibited an activity peak at the same time point, we investigated whether the centrosomal localization of TUFT1 is regulated by NEK2-mediated phosphorylation. To this end, we constructed a phospho-mimetic mutant of TUFT1 (TUFT1-2D) and compared the binding affinity of the TUFT1 mutants to C-Nap1. Co-immunoprecipitation assays revealed that TUFT1-2A displayed increased binding affinity for C-Nap1, whereas TUFT1-2D had the opposite effect (Fig. 5E, F). Accordingly, the centrosomal localization of TUFT1-2D was weaker than that of TUFT1-WT and TUFT1-2A (Fig. 5G). Additionally, the disassociation of TUFT1 from the centrosome upon mitotic entry was significantly inhibited by NEK2 depletion (Fig. 5H–J). Taken together, these results indicate that NEK2-mediated phosphorylation weakens the interaction between TUFT1 and C-Nap1 and promotes the removal of TUFT1 from the centrosome upon entry into mitosis. Phosphorylation of TUFT1 did not affect its interaction with PLK1 (Fig. S4C).

### Phosphorylation status of TUFT1 coordinates centrosome number and cell proliferation in cervical and breast cancers

We further investigated whether NEK2-mediated phosphorylation affects the biological functions of TUFT1. We mainly focused on cervical and breast cancers for subsequent investigations because both NEK2 and TUFT1 have been reported to promote the progression and chemoresistance of these two cancers [38–40, 44–48].

First, we depleted endogenous TUFT1 in MDA-MB-231 cells using siRNAs and found that the proportion of mitotic cells with multipolar spindles and interphase cells with amplified centrioles significantly increased following TUFT1 knockdown (Fig. S5A–S5F). All phenotypes were rescued by transient siRNA-resistant exogenous TUFT1 transfection (Fig. S5A–S5F), indicating that TUFT1 suppresses CA in breast cancer cells. To further confirm these effects, we generated a TUFT1-knockout MDA-MB-231 cell line and validated its knockout efficiency (Fig. S6A–S6D). Similar phenotypes were observed in the TUFT1-knockout MDA-MB-231 cells, which were rescued by the reintroduction of exogenous TUFT1 (Fig. S6C, S6E–S6I). These results indicate that TUFT1 suppresses CA and mitotic spindle multipolarity in both cervical and breast cancer cells.

**Fig. 6:**
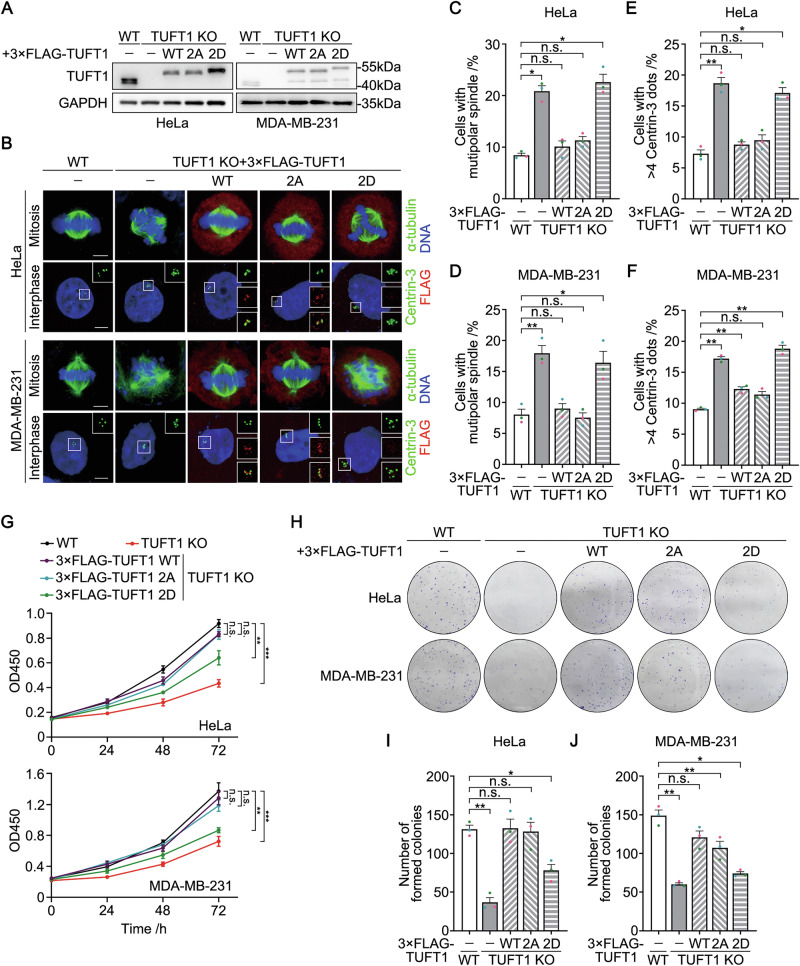
Phosphorylation status of TUFT1 coordinates centrosome number and cell proliferation in cervical and breast cancers. **A** Immunoblots of TUFT1 in wild-type (WT) and TUFT1-knockout (KO) HeLa and MDA-MB-231 cells that stably express 3×FLAG-TUFT1 (WT, 2A, or 2D). GAPDH served as a loading control. **B** Immunostaining of α-tubulin (green, upper panel) or Centrin-3 (green, bottom panel) in WT and TUFT1-KO HeLa and MDA-MB-231 cells that stably express 3×FLAG-TUFT1 (WT, 2A, or 2D) (red). DNA was stained with DAPI (blue). Scale bar, 5 μm. **C**, **D** Quantification of cells with multipolar spindle in (**B**) (*n* > 300 cells from three independent experiments). **E**, **F** Quantification of Centrin-3 foci number in (**B**) (*n* > 300 cells from three independent experiments). **G** CCK-8 assays of WT and TUFT1-KO HeLa and MDA-MB-231 cells that stably express 3×FLAG-TUFT1 (WT, 2A, or 2D) (three independent experiments). **H** Colony formation assays of WT and TUFT1-KO HeLa and MDA-MB-231 cells that stably express 3×FLAG-TUFT1 (WT, 2A, or 2D). **I**, **J** Quantification of the number of colonies in (**H**) (three independent experiments). Data in (**C**–**F**), (**G**), (**I**), and (**J**) are shown as the mean ± s.e.m. *P*-values are shown in the figure (one-way ANOVA).

We further generated TUFT1-knockout HeLa and MDA-MB-231 cell lines stably expressing exogenous TUFT1 (WT, 2A, or 2D) (Fig. 6A). First, we investigated whether the phosphorylation status of TUFT1 affects the centrosome number. Compared to TUFT1-WT and TUFT1-2A, TUFT1-2D resulted in markedly increased rates of CA and spindle multipolarity (Fig. 6B–F), indicating that continuous phosphorylation of TUFT1 favors the occurrence of CA. CCK-8 and colony formation assays indicated that proliferation of cells expressing TUFT1-2D was considerably inhibited, whereas that of cells expressing TUFT1-2A was similar to that of cells expressing TUFT1-WT (Fig. 6G–J), indicating that continuous dephosphorylation of TUFT1 favors cell proliferation.

**Fig. 7:**
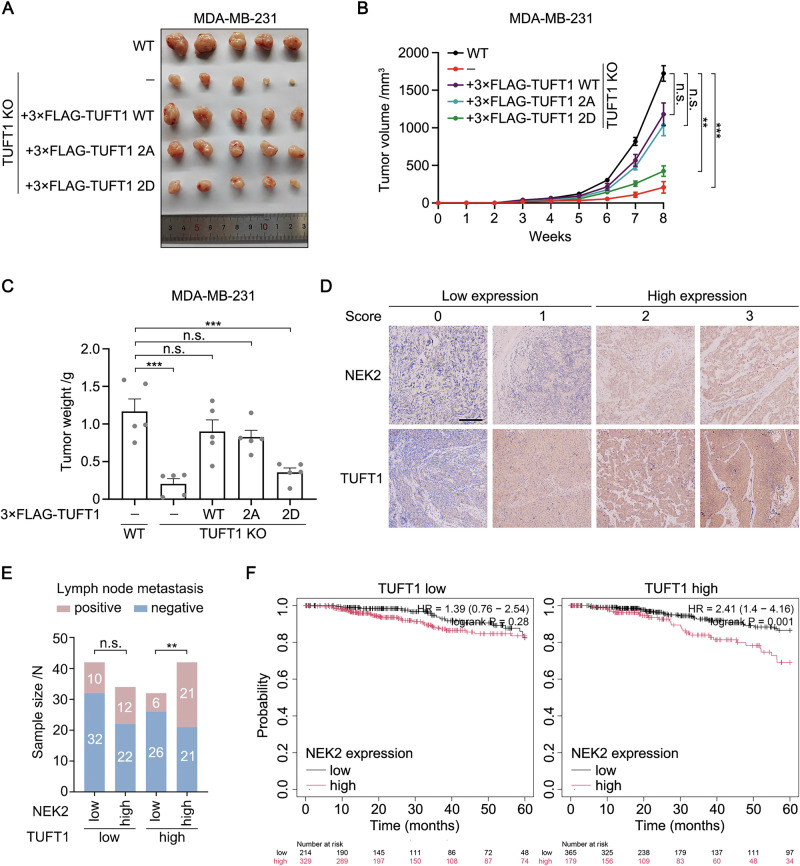
Upregulation of the NEK2/TUFT1 axis is associated with poor outcome in breast cancer. **A** Wild-type (WT) and TUFT1-knockout (KO) MDA-MB-231 cells that stably express 3×FLAG-TUFT1 (WT, 2A, or 2D) were injected into mammary fat pads of female nude mice (*n* = 5). Representative photograph of excised tumors is shown. **B** Quantification of the tumor volume in (**A**). **C** Quantification of the tumor weight in (**A**). **D** Representative images of immunohistochemical staining of NEK2 and TUFT1. Scale bar, 200 μm. **E** Quantification of the number of lymph node metastasis in each subgroup, using the patient cohort in (**D**). **F** Correlation between overall survival and NEK2/TUFT1 expression in breast cancer. Data in (**B**) and (**C**) are presented as mean ± s.e.m. *P*-values are shown in the figure (one-way ANOVA in (**B**) and (**C**); chi-square test in (**E**); and two-sided log-rank test in (**F**)).

To test the effects of TUFT1 phosphorylation on in vivo tumor growth, we injected all the MDA-MB-231 cell lines mentioned above into the mammary fat pads of female nude mice, and monitored tumor growth. Consistent with the results from the cultured cells, the volume and weight of tumors originating from TUFT1-2D cells were markedly lower than those originating from TUFT1-WT and TUFT1-2A cells (Fig. 7A–C). In addition, HeLa cell lines corresponding to TUFT1 mutations were subcutaneously injected into female nude mice to induce ectopic tumor formation. We also observed retarded tumor growth in the TUFT1-2D group (Fig. S7A–S7C). These results suggest that sustained TUFT1 phosphorylation inhibits cervical and breast cancer cell growth in vivo. Overall, the phosphorylation status of TUFT1 is essential for coordinating centrosome number and cell proliferation in cervical and breast cancers.

### Upregulation of the NEK2/TUFT1 axis is associated with poor outcome in breast cancer

Next, we sought to investigate the effects of TUFT1 phosphorylation on clinical outcome in patients with breast cancer. We performed immunohistochemical analysis of triple-negative breast cancer (TNBC) tissue microarrays and assessed the correlation between NEK2/TUFT1 expression and patient outcome. Each sample was scored on a scale of 0–3 based on the percentage of immunoreactive cells and the staining intensity, and the samples were classified as low (0–1) and high (2–3) expression groups (Fig. 7D). We found that patients with high NEK2 and TUFT1 expression had a significantly high rate of lymph node metastasis (Fig. 7E and Table S1), reflecting poor outcome. We further assessed the correlation between NEK2/TUFT1 expression and the overall survival (OS) of patients with breast cancer based on Kaplan-Meier Plotter databases. NEK2 expression negatively correlated with OS only in the TUFT1 high-expression group (Fig. 7F), indicating that NEK2 drives breast cancer progression by regulating TUFT1. Meanwhile, within the TUFT1 high-expression group, patients with hyperphosphorylated TUFT1 exhibited reduced OS (Fig. 7F), implying that for the entire breast cancer cell population, the disadvantage of TUFT1 hyperphosphorylation on cell proliferation may be overcome by its advantage of increasing CA and CIN. A similar conclusion was reached for uterine corpus endometrial and thyroid carcinomas (Fig. S7D and S7E). Contrastingly, in some other cancers, such as stomach adenocarcinoma and head-neck squamous cell carcinoma, patients with hypophosphorylated TUFT1 exhibited reduced OS (Fig. S7F and S7G). This may be because cell proliferation generally precedes genetic variations in these cancers, which require the dephosphorylation of TUFT1. In summary, upregulation of the NEK2/TUFT1 axis predicts poor outcome in patients with breast cancer.

## Discussion

**Fig. 8:**
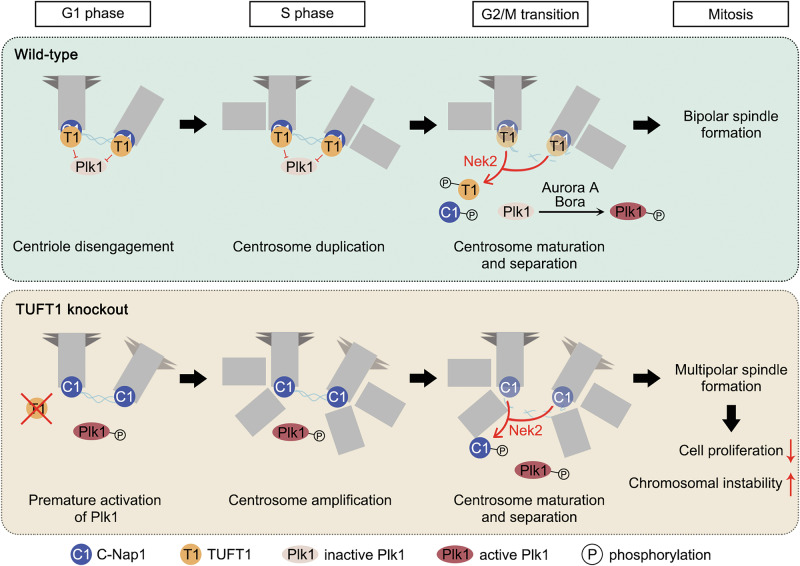
Schematic model. TUFT1 localizes to the centrosome and prevents CA by blocking PLK1 activity. At the onset of mitosis, NEK2-mediated phosphorylation promotes the removal of TUFT1 from the centrosome. The loss of TUFT1 induces premature activation of PLK1, triggering CA and mitotic abnormalities, thereby suppressing cell proliferation and increasing CIN.

Recent studies have shown that TUFT1 is an oncoprotein that promotes proliferation, metastasis, and drug resistance in breast cancer cells and is associated with worse clinical status and poor prognosis [38–40]. In this study, we identified a novel mechanism of TUFT1 in the regulation of breast cancer progression. TUFT1 localizes to the centrosome and prevents CA by blocking PLK1 activity. At the onset of mitosis, NEK2-mediated phosphorylation promotes the removal of TUFT1 from the centrosome. The loss of TUFT1 induces premature activation of PLK1, triggering CA and mitotic abnormalities, thereby suppressing cell proliferation and increasing CIN (Fig. 8). Besides, TUFT1 activates the Rab5/Rac1/β-catenin pathway and concurrently downregulates the NF-κB pathway and proapoptotic factors, thus promoting metastasis, stemness, and chemoresistance of TNBC cells [38, 39]. In addition, TUFT1 promotes malignancy in TNBC by upregulating the expression of the lncRNA DANCR, which functions as a miR-874-3p sponge that modulates SOX2 positively [40]. Overall, TUFT1 exerts oncogenic roles through multiple mechanisms in breast cancer.

It is well-established that CA has contradictory effects on cancer cell survival. CA-mediated mitotic spindle abnormalities are considered as a primary source of CIN via increased frequency of chromosome mis-segregation and DNA breaks [49, 50]. CIN further drives the diversification of tumor subclones by increasing intratumor genetic heterogeneity within the population and accelerates evolutionary adaptations under selective pressures [6]. However, accumulation of excess mature centrosomes triggers the formation of the PIDDosome, resulting in p53-dependent cell-cycle arrest and cell death [7]. In this study, we found that centrosome number is regulated by NEK2-mediated phosphorylation of TUFT1. Hyperphosphorylation of TUFT1 favors the occurrence of CA and inhibits cell proliferation, whereas hypophosphorylation of TUFT1 has the opposite effect. Thus, TUFT1 is a crucial module that balances cell proliferation and genetic variation in cancer cells. In general, patients with breast cancer with hyperphosphorylated TUFT1 exhibited an increased rate of lymph node metastasis and reduced OS (Fig. 7E, F), consistent with the consensus that high levels of CIN are related to poor clinical outcome. Our results also highlight the importance of post-translational modifications in regulating the biological functions of TUFT1. We previously reported that SUMOylation of TUFT1 is essential for AKT/mTOR pathway activation in gastric cancer cells [51]. Whether this modification also occurs in other cancer cells, and the crosstalk between SUMOylation and phosphorylation, requires further investigation.

PLK1 is activated in late G2 phase and mitosis, whereas premature activation of PLK1 in early phases has been shown to induce premature centriole disengagement and centriole reduplication [15, 18–21]. Our results suggest that the loss of TUFT1 leads to premature activation of PLK1, whereas overexpression of TUFT1 decreases PLK1 activity (Figs. 4A–J and S3A–S3F), suggesting that TUFT1 negatively regulates PLK1 activity. At least two possible mechanisms may account for this. First, Aurora A phosphorylates and activates PLK1 in late G2 phase [52, 53], and TUFT1 may interfere the interaction between Aurora A and PLK1. Second, dephosphorylation of PLK1 is dependent on ATM/Chk activation and PP2A activity [54, 55], and TUFT1 may promote PLK1 dephosphorylation by modulating these pathways. Apart from TUFT1, several other proteins are also reported to regulate centrosome duplication via PLK1, such as BUB1-related protein 1, liver kinase B1, and receptor of activated protein C kinase 1 [20, 56, 57]. Whether any synergistic or antagonistic effect exists among these proteins requires further studies. In conclusion, our results illustrate the essential role of TUFT1 in the regulation of tumor progression through centrosome number control, implying that TUFT1 may be a promising target for diagnostic and therapeutic approaches for cancers.

## Materials and methods

### Plasmid construction

TUFT1 (NM_020127.3) and its mutants were amplified by PCR and cloned into p3×FLAG-CMV-7.1 (Sigma-Aldrich, MO, USA), pEGFP-C1 (Clontech Laboratories, CA, USA), and pCDH-CMV-MCS-EF1-Puro (System Biosciences, CA, USA). C-Nap1 (NM_007186.5) was amplified by PCR and cloned into pEGFP-N3 (Clontech Laboratories). PLK1 (NM_005030.6) and its mutants were amplified by PCR and cloned into the pEGFP-C1. NEK2 (NM_002497.3) was amplified by PCR and cloned into p3×FLAG-CMV-7.1 and pEGFP-C1.

### Antibodies

The following primary antibodies were used in this study: rabbit anti-TUFT1 (23385-1-AP, Proteintech, Wuhan, China), mouse anti-Centrin-3 (SC-100933, Santa Cruz Biotechnology, TX, USA), mouse anti-SAS-6 (SC-81431, Santa Cruz Biotechnology), mouse anti-C-Nap1 (AG4725, Beyotime Biotechnology, Shanghai, China), mouse anti-FLAG (F1804, Sigma-Aldrich), mouse anti-GFP (66002-1-Ig, Proteintech), mouse anti-α-tubulin (T6199, Sigma-Aldrich), mouse anti-γ-tubulin (T6557, Sigma-Aldrich), rabbit anti-Cep97 (22050-1-AP, Proteintech), rabbit anti-PLK4 (28750-1-AP, Proteintech), mouse anti-GAPDH (60004-1-Ig, Proteintech), rabbit anti-FLAG (20543-1-AP, Proteintech), rabbit anti-γ-tubulin (T3559, Sigma-Aldrich), mouse anti-PLK1 (ab17056, Abcam, Cambridge, UK), rabbit anti-PLK1 (4513, Cell Signaling Technology, MA, USA), rabbit anti-PLK1 (phospho T210) (ab155095, Abcam), mouse anti-STIL (66876-1-Ig, Proteintech), rabbit anti-Cep152 (21815-1-AP, Proteintech), rabbit anti-Cep192 (18832-1-AP, Proteintech), rabbit anti-Cep164 (22227-1-AP, Proteintech), rabbit anti-Odf2 (12058-1-AP, Proteintech), rabbit anti-Cyclin B1 (12231, Cell Signaling Technology), and mouse anti-NEK2 (610593, BD Biosciences, NJ, USA). Secondary antibodies used for immunoblotting were HRP-conjugated goat anti-mouse/rabbit IgG (H+L) (SA00001-1, SA00001-2, Proteintech). Secondary antibodies used for confocal microscopy and 3D-SIM were CoraLite^®^488/594/647-conjugated goat anti-mouse/rabbit IgG (SA00013-1, SA00013-2, SA00013-3, SA00013-4, SA00014-10, Proteintech).

### RNA interference

The following oligonucleotide sequences were used for siRNA-mediated depletion of proteins: human TUFT1 siRNA-1, 5′-GGAUAUAAGUAGCAAGCUUGA-3′; human TUFT1 siRNA-2, 5′-GUAGCCUUUUGCGGAAAAAUU-3′; human C-Nap1 siRNA, 5′-UUCUCCGAACGUGUCACGU-3′; and human NEK2 siRNA, 5′-AAACAUCGUUCGUUACUAU-3′. The sequence of negative control siRNA was 5′-UUCUCCGAACGUGUGUCACGU-3′.

The siRNA-resistant cDNA was cloned using PCR. The siRNA-targeted regions of TUFT1 were mutated to 5′-CGCGAAGACATTAGCAGTAAACTA-3′.

### Cell culture, transfection, and chemical treatment

HEK293T, HeLa, and MDA-MB-231 cell lines were purchased from Pricella Biotechnology (Wuhan, China) and were cultured in DMEM (BasalMedia, Shanghai, China) containing 10% FBS (Sbjbio, Nanjing, China) in an incubator at 37 °C with 5% CO_2_. JetPRIME^®^ reagent (Polyplus, Illkirch, France) was used for plasmid and siRNA transfection.

For G1-, S-, G2- and M-phase synchronization, HeLa cells were treated with 0.4 mM mimosine (HY-N0928, MedChemExpress, NJ, USA), 2.5 mM thymidine (HY-N1150, MedChemExpress), 15 μM RO-3306 (HY-12529, MedChemExpress), or 100 ng/mL nocodazole (HY-13520, MedChemExpress) for 16 h, respectively, and then released. For inhibition of PLK1, HeLa cells were treated with 10 nM volasertib (S2235, Selleck, TX, USA) for 48 h.

### Generation of TUFT1-knockout cell lines

TUFT1-knockout HeLa and MDA-MB-231 cell lines were generated as previously described [51]. The sequence of the target oligo was 5′-CACCGGGAGTCCCATGATGGACATG-3′.

### Immunofluorescence microscopy

Cells were fixed and permeabilized in methanol for 10 min at –20 °C, incubated with primary antibodies in 5% BSA overnight at 4 °C and then incubated with secondary antibodies for 1 h at room temperature (RT). DNA was stained with 1 μg/mL 4′,6-diamidino-2-phenylindole (DAPI).

For confocal microscopy, samples were observed at RT using a TCS SP8 microscope (Leica, Wetzlar, Germany) equipped with a 63×/1.40 NA objective lens (Leica), and images were captured using LAS X software (Leica). For 3D-SIM, samples were observed at RT using an Elyra 7 system (Zeiss, Oberkochen, Germany) equipped with a 63×/1.40 NA objective lens (Zeiss). In the Lattice SIM mode, we captured a 3 μm Z-stack at 0.125 μm interval for all channels using the ZEN Black software (Zeiss). Raw images were processed using the 3D-SIM² method with ‘Standard–Fixed’ settings. The images were further subjected to maximum intensity projection to convert the stacked images into a single image.

### Immunoblotting

Protein samples were separated by SDS-PAGE and then transferred to PVDF membranes (Merck Millipore, MA, USA). Membranes were blocked with 4% non-fat milk for 1 h at RT and incubated with the indicated primary antibodies and HRP-conjugated secondary antibodies. All membranes were analyzed using an automated chemiluminescence immunoassay analyzer (Tanon, Shanghai, China). The original Western blots can be found in the Supplementary Materials.

### Immunoprecipitation assays

The cells were lysed in lysis buffer (150 mM NaCl, 50 mM HEPES, 1 mM MgCl_2_, 1 mM EGTA, 0.5% Triton X-100, and protease inhibitors; pH 7.4). After centrifugation, the supernatant was mixed with the indicated antibodies and protein G-Sepharose beads (GE Healthcare, IL, USA) at 4 °C for 4 h or overnight. The beads were washed with lysis buffer, then boiled for 10 min at 100 °C in protein loading buffer.

### Flow cytometry analysis

Cells were collected and centrifuged at 1000 × *g* for 5 min. After washing twice with cold PBS, cells were fixed in cold 70% ethanol for 2 h. Fixed cells were washed twice with PBS and treated with RNase A and propidium iodide for 30 min at 37 °C, then analyzed using LSRFortessa™ (BD Biosciences).

### Generation of stable expression cell lines

HEK293T cells were co-transfected with psPAX.2, pCMV-VSV-G, and pCDH-CMV-MCS-EF1-3×FLAG-TUFT1 (WT, 2A, or 2D) plasmids for 72 h. The culture supernatant was passed through a 0.22-μm filter, and the packaged lentivirus was concentrated using 10% PEG 8000. TUFT1-knockout cells were infected with the lentivirus in the presence of 8 μg/mL polybrene (E1299, Selleck), and puromycin-resistant cells were selected and cultured.

### Cell proliferation assays

For CCK-8 assays, cells (3 × 10^3^ cells/well) were seeded into 96-well plates. CCK-8 reagent (10 μL; MedChemExpress) was added to each well and the plates were incubated at 37 °C for 1 h before measuring absorbance at a wavelength of 450 nm. For colony formation assays, cells (500 cells/well) were seeded in 6-well plates. Cells were maintained in culture medium for 14 days, followed by staining with crystal violet.

### Animal experiments

All experimental procedures were approved by the Animal Ethics Committee of Central Hospital Affiliated to Shandong First Medical University (#JNCHIACUC2024-46). BALB/c nude mice (female, 5–6 weeks old) were randomly divided into 10 groups (*n* = 5) and housed in a temperature-controlled environment with 12 h light/12 h dark cycle. MDA-MB-231 cells (2 × 10^6^; WT, TUFT1-KO, or stable cell lines) suspended in 100 μL of 1:1 (v/v) PBS and ABW^®^ Matrigengel (ABWbio, Shanghai, China) were injected into mammary fat pads of the mice. HeLa cells (2 × 10^6^; WT, TUFT1-KO, or stable cell lines) suspended in 100 μL of PBS were subcutaneously injected into right flanks of the mice. Tumor volumes were measured with a Vernier caliper every 3 days and calculated as length × width^2^ × 0.5. After 8 weeks, the mice were sacrificed and the sub-skin grafts were dissected and weighed.

### Immunohistochemical staining

Immunohistochemical staining was performed as described previously [58] on breast cancer tissue microarrays (#HBreD180Bc01-2; Shanghai Outdo Biotech Company, China) with anti-NEK2 and anti-TUFT1 antibodies. Two observers independently scored the tissues on a scale of 0–3 based on the percentage of immunoreactive cells and staining intensity. The study was performed in accordance with the Declaration of Helsinki. All samples were collected after obtaining written informed consent from the participants. The research protocol was approved by the Ethics Committee of Shanghai Outdo Biotech Company (#SHYJS-CP-2210009).

### Overall survival analysis

The Kaplan–Meier Plotter (https://www.kmplot.com/analysis/) [59] is an online tool that provides gene survival analysis in various cancer types. The specimens of indicated cancer types were filtered by the median expression of TUFT1, wherein expression levels above the median were classified as high expression and those below as low expression. Subsequently, the overall survival analysis of NEK2 expression was performed independently in both subgroups.

### Measurements and statistical analyses

The intensity of the immunoblot bands and fluorescent signals were measured using ImageJ software (NIH). Images were imported into ImageJ as grayscale TIFF files. Regions of interest (ROIs) corresponding to immunoblot bands or centrosome areas were defined manually. Background signal intensity was quantified from at least three band- or cell-free regions per image, and the mean background intensity was subtracted from all ROI measurements. The immunoblot band or fluorescent signal within each ROI was quantified using ImageJ’s measurement function, reporting integrated density. Data were normalized to relevant control groups. GraphPad Prism 8 software (San Diego, CA, USA) was used for statistical analyses. Each experiment was independently repeated three times to ensure reliability. Student’s *t*-test was employed to compare differences between two groups. One-way ANOVA was employed to evaluate differences among multiple groups. For categorical variables, chi-square test was applied. Overall survival analysis was performed using the Kaplan–Meier method. *P* value < 0.05 was considered statistically significant. Asterisks indicate corresponding statistical significance: ***P < 0.001; **P < 0.01; *P < 0.05. The statistical details of each experiment including the statistical significance and *n* values are provided in the figure legends [60].

## Supplementary information

Supplemental Figure and Table

Original Western blots

## Data Availability

The data supporting the findings of this study are available from the corresponding authors upon request.
