# Supplementary material for: TUFT1 regulates cancer progression by suppressing centrosome amplification and mitotic spindle multipolarity
Source: Cell Death Dis. 2025 Sep 29;16(1):673. doi: 10.1038/s41419-025-08010-3 (PMC12480466; doi:10.1038/s41419-025-08010-3)

**Supplementary figures**


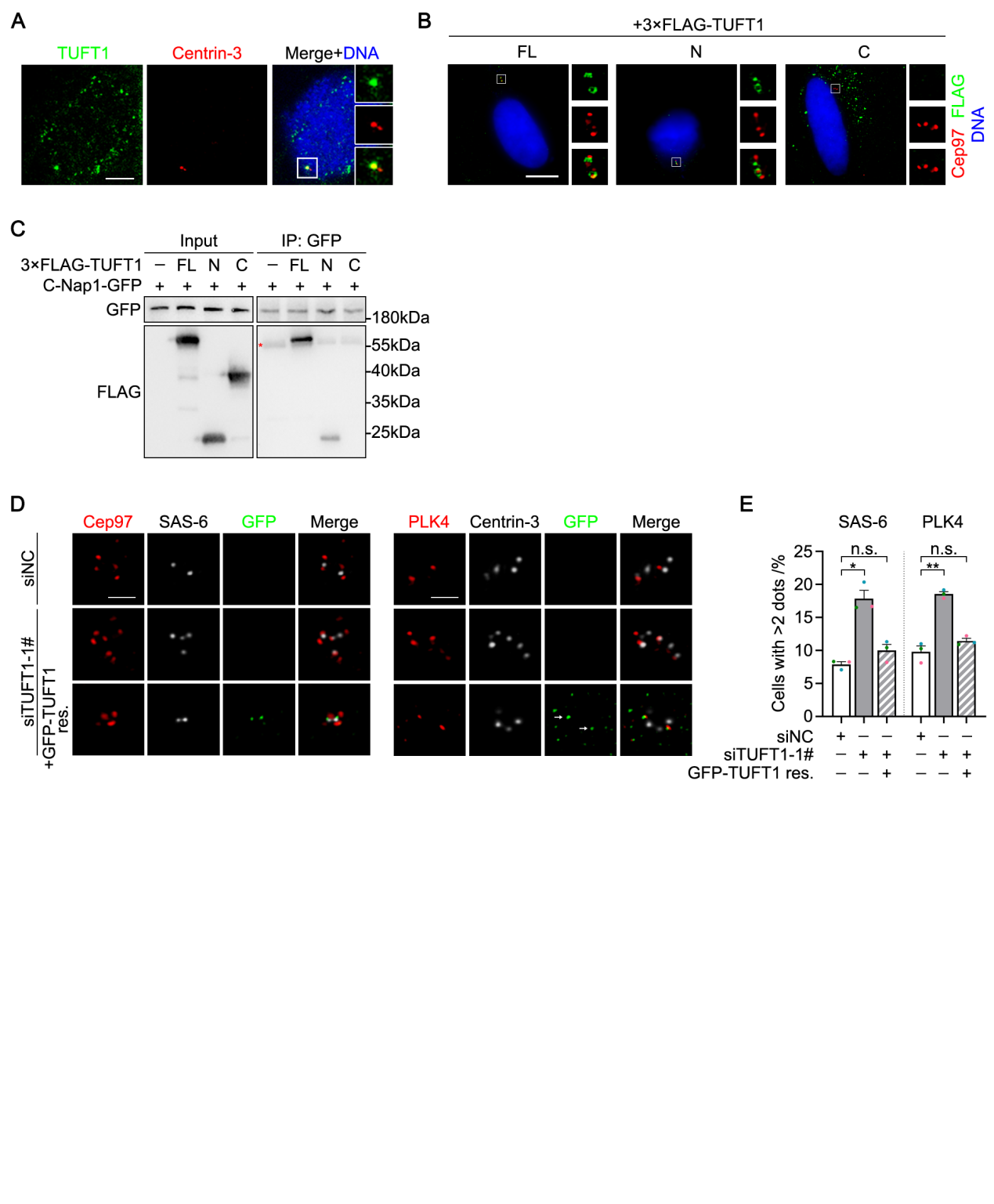


**Figure S1. TUFT1 localizes to the centrosome through its N-terminus.** (A) Immunostaining of TUFT1 (green) and Centrin-3 (red) in HeLa cells. DNA was stained with 4′,6-diamidino-2-phenylindole (DAPI) (blue). Scale bar, 5 μm. (B) Immunostaining of Cep97 (red) in HeLa cells overexpressing 3×FLAG-TUFT1 (FL, N, or C) (green). DNA was stained with DAPI (blue). Scale bar, 10 μm. (C) Lysates of HEK293T cells overexpressing C-Nap1-GFP and 3×FLAG-TUFT1 (FL, N, or C) were subjected to immunoprecipitation (IP) and immunoblotting, as indicated. The red asterisk (*) indicates IgG heavy chains. (D) Immunostaining of Cep97 (red, left panel), SAS-6 (white, left panel), PLK4 (red, right panel), and Centrin-3 (white, right panel) in HeLa cells transfected with negative control (NC)- or TUFT1-siRNA. SiRNA-resistant GFP-TUFT1 (green) was transiently transfected into knockdown cells to rescue the phenotype. Arrows: centrosome-localized exogenous TUFT1. Scale bar: 1 μm. (E) Quantification of SAS-6 and PLK4 foci numbers in (D) (n > 300 cells from three individual experiments). Data are shown as the mean ± s.e.m. *P*-values are shown in the figure (one-way ANOVA).


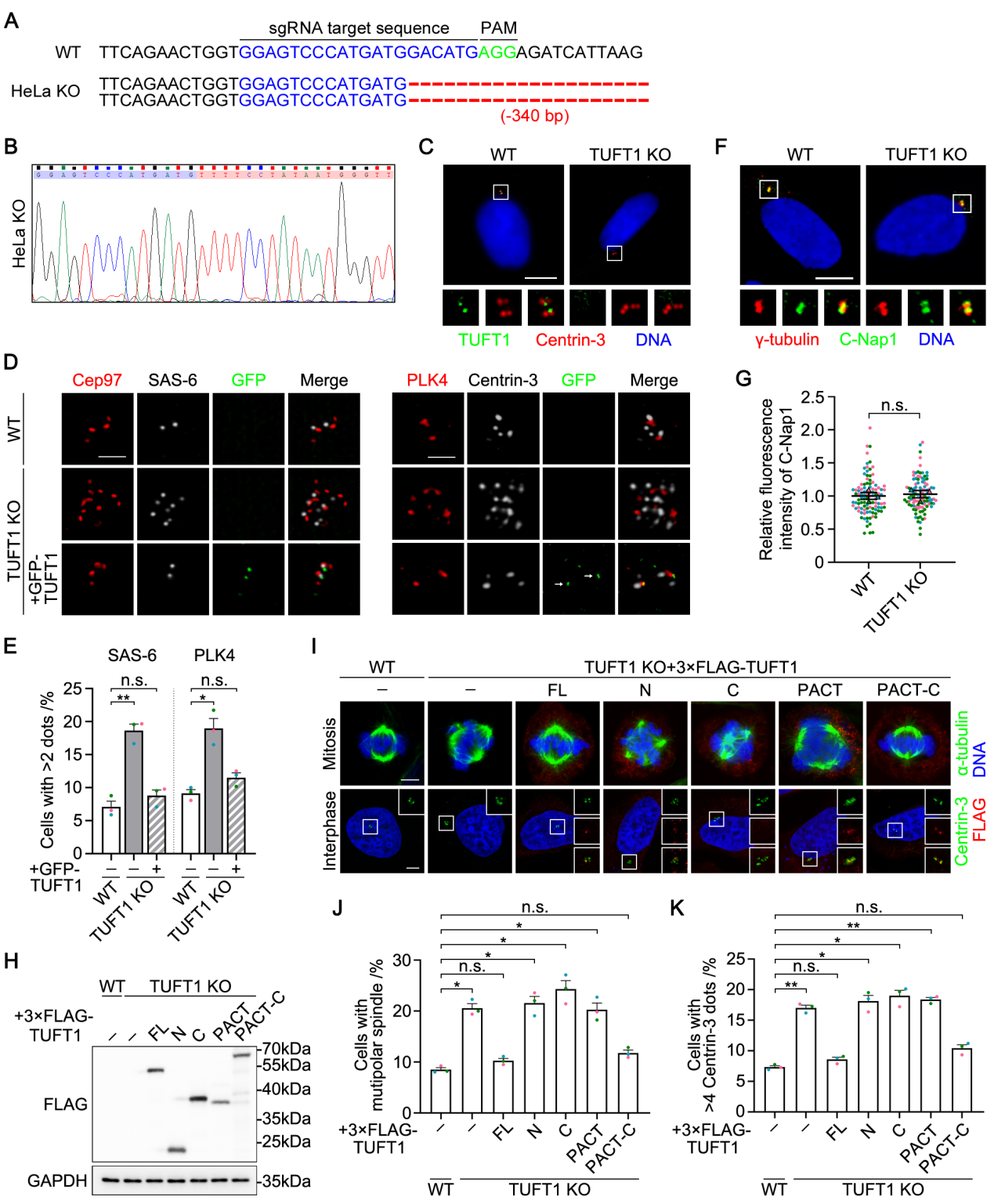


**Figure S2. TUFT1 knockout induces centrosome amplification and mitotic spindle multipolarity in HeLa cells.** (A) Schematic of the TUFT1-knockout (KO) HeLa cell line. The protospacer adjacent motif (PAM) sequence is highlighted in green, single guide RNA (sgRNA) target sequence is highlighted in blue, and mutated sequence is highlighted in red. (B) Genome sequence of the KO cell line around the targeted region. (C) Immunostaining of TUFT1 (green) and Centrin-3 (red) in wild-type (WT) and TUFT1-KO HeLa cells. DNA was stained with DAPI (blue). Scale bar, 10 μm. (D) Immunostaining of Cep97 (red, left panel), SAS-6 (white, left panel), PLK4 (red, right panel), and Centrin-3 (white, right panel) in WT and TUFT1-KO HeLa cells. GFP-TUFT1 (green) was transiently transfected into KO cells to rescue the phenotype. Arrows: centrosome-localized exogenous TUFT1. Scale bar: 1 μm. (E) Quantification of SAS-6 and PLK4 foci numbers in (D) (n > 300 cells from three individual experiments). (F) Immunostaining of C-Nap1 (green) and γ-tubulin (red) in WT and TUFT1-KO HeLa cells. DNA was stained with DAPI (blue). Scale bar, 10 μm. (G) Quantification of the fluorescence intensity of C-Nap1 in (F) (n > 100 cells from three independent experiments). (H) Immunoblots of WT and TUFT1-KO HeLa cells transfected with 3×FLAG-TUFT1 (FL, N, C, and PACT-C) and the PACT domain. GAPDH served as a loading control. (I) Immunostaining of α-tubulin (green, upper panel) or Centrin-3 (green, bottom panel) in WT or TUFT1-KO HeLa cells. 3×FLAG-TUFT1 (FL and mutants) or the PACT domain was transiently transfected into KO cells to rescue the phenotype. DNA was stained with DAPI (blue). Scale bar, 5 μm. (J) Quantification of cells with multipolar spindle in (I) (n > 300 cells from three independent experiments). (K) Quantification of Centrin-3 foci number in (I) (n > 300 cells from three independent experiments). Data in (E), (G), (J), and (K) are shown as the mean ± s.e.m. *P*-values are shown in the figure (paired two-tailed Student’s *t*-test in (G); one-way ANOVA in (E), (J) and (K)).


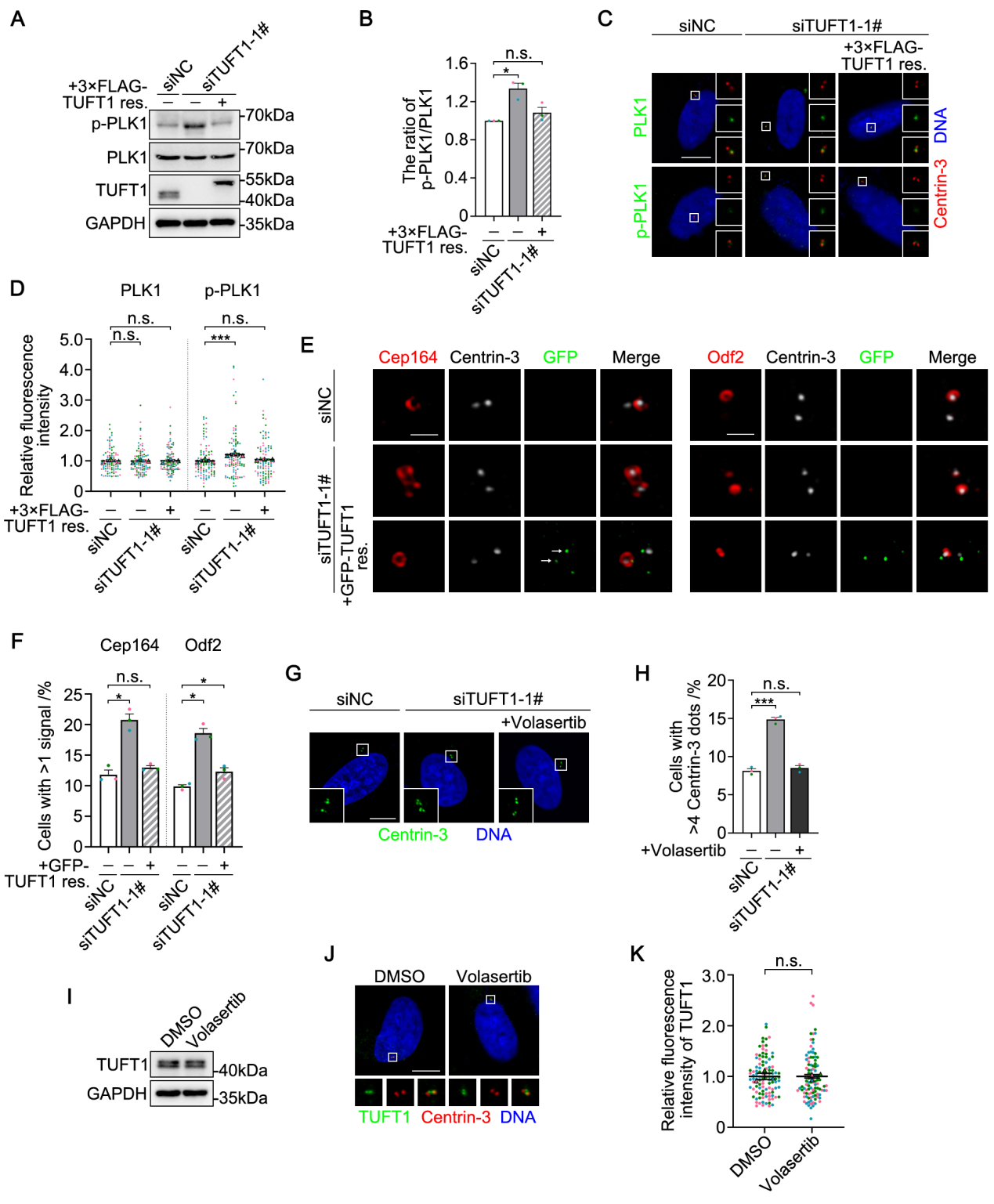


**Figure S3. TUFT1** **knockdown hyperactivates PLK1.** (A) Evaluation of the effects of TUFT1 knockdown on PLK1 activation in HeLa cells by immunoblotting. Phospho-PLK1 (p-PLK1) indicates PLK1 activation. SiRNA-resistant 3×FLAG-TUFT1 was transiently transfected into knockdown cells to rescue the phenotype. GAPDH served as a loading control. (B) Quantification of the ratio of p-PLK1/PLK1 in (A) (three independent experiments). (C) Immunostaining of PLK1 (green, upper panel) or p-PLK1 (green, bottom panel) and Centrin-3 (red) in HeLa cells transfected with negative control (NC)- or TUFT1-siRNA. SiRNA-resistant 3×FLAG-TUFT1 was transiently transfected into knockdown cells to rescue the phenotype. DNA was stained with DAPI (blue). Scale bar, 10 μm. (D) Quantification of the fluorescence intensities of PLK1 and p-PLK1 in (C) (n > 100 cells from three independent experiments). (E) Immunostaining of Cep164 (red, left panel), Odf2 (red, right panel), and Centrin-3 (white) in HeLa cells transfected with NC- or TUFT1-siRNA. SiRNA-resistant GFP-TUFT1 (green) was transiently transfected into knockdown cells to rescue the phenotype. Arrows: centrosome-localized exogenous TUFT1. Scale bar: 1 μm. (F) Quantification of Cep164 and Odf2 signal numbers in (E) (n > 300 cells from three individual experiments). (G) Immunostaining of Centrin-3 (green) in HeLa cells transfected with NC- or TUFT1-siRNA. Knockdown cells were treated with or without volasertib. DNA was stained with DAPI (blue). Scale bar, 10 μm. (H) Quantification of Centrin-3 foci number in (G) (n > 300 cells from three independent experiments). (I) Immunoblots of TUFT1 in HeLa cells treated with or without volasertib. GAPDH served as a loading control. (J) Immunostaining of TUFT1 (green) and Centrin-3 (red) in HeLa cells treated with or without volasertib. DNA was stained with DAPI (blue). Scale bar, 10 μm. (K) Quantification of the fluorescence intensity of TUFT1 in (J) (n > 100 cells from three independent experiments). Data in (B), (D), (F), (H) and (K) are shown as the mean ± s.e.m. *P*-values are shown in the figure (one-way ANOVA in (B), (D), (F) and (H); paired two-tailed Student’s *t*-test in (K)).


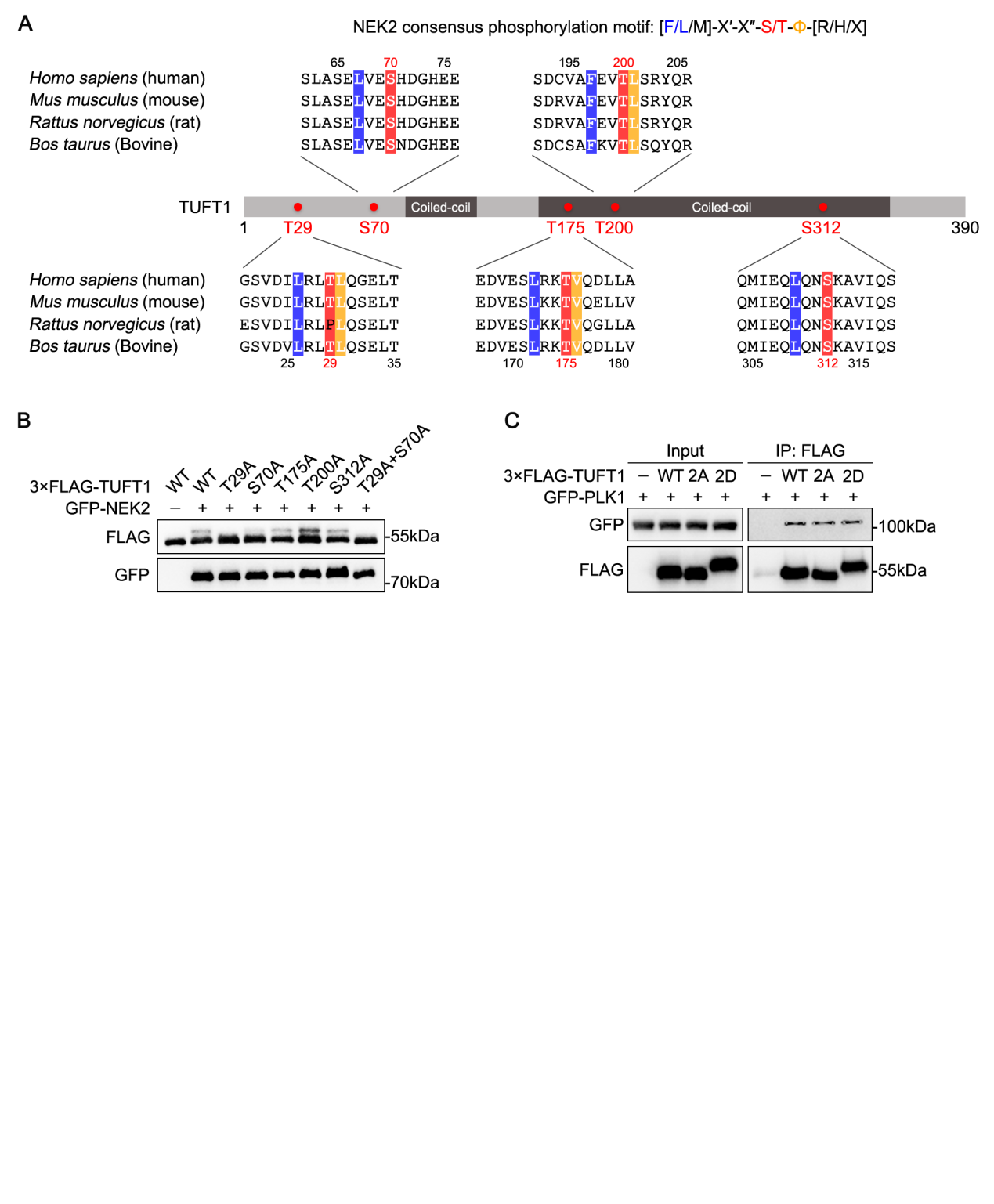


**Figure S4. TUFT1 is phosphorylated by NEK2 at T29 and S70.** (A) Schematic of NEK2 consensus phosphorylation motif. Within the indicated motif, both X’ and X’’ denote any amino acid except proline, and X’ also includes some positive selection for basic and hydrophobic residues; F denotes any hydrophobic amino acid with proline, asparticacid, and glutamicacid excluded from this position; and X denotes any amino acid [43]. The amino acid sequences of wild-type (WT) TUFT1 among 4 species is shown below. Possible phosphorylation sites are highlighted in red. (B) Immunoblots of HEK293T cells overexpressing GFP-NEK2 and 3×FLAG-TUFT1 (WT and mutants). (C) Lysates of HEK293T cells overexpressing GFP-PLK1 and 3×FLAG-TUFT1 (WT, 2A, or 2D) were subjected to immunoprecipitation (IP) and immunoblotting, as indicated.


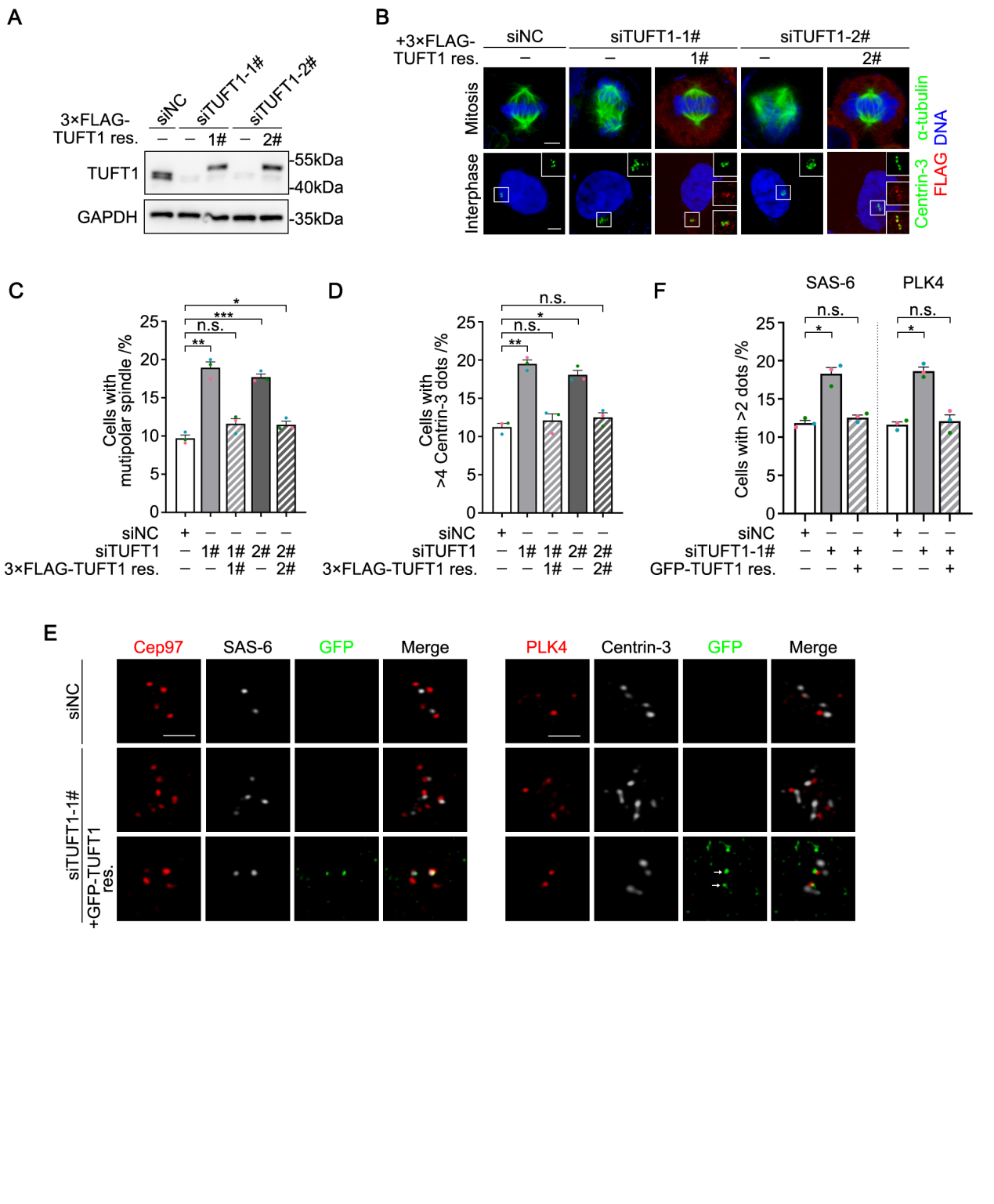


**Figure S5. TUFT1 knockdown induces centrosome amplification and mitotic spindle multipolarity in MDA-MB-231 cells.** (A) Immunoblots of TUFT1 in MDA-MB-231 cells transfected with negative control (NC)- or TUFT1-siRNA. SiRNA-resistant 3×FLAG-TUFT1 was transiently transfected into knockdown cells to rescue the phenotype. GAPDH served as a loading control. (B) Immunostaining of α-tubulin (green, upper panel) or Centrin-3 (green, bottom panel) in MDA-MB-231 cells transfected with NC- or TUFT1-siRNA. SiRNA-resistant 3×FLAG-TUFT1 (red) was transiently transfected into knockdown cells to rescue the phenotype. DNA was stained with DAPI (blue). Scale bar, 5 μm. (C) Quantification of cells with multipolar spindle in (B) (n > 300 cells from three independent experiments). (D) Quantification of Centrin-3 foci number in (B) (n > 300 cells from three independent experiments). (E) Immunostaining of Cep97 (red, left panel), SAS-6 (white, left panel), PLK4 (red, right panel), and Centrin-3 (white, right panel) in MDA-MB-231 cells transfected with NC- or TUFT1-siRNA. SiRNA-resistant GFP-TUFT1 (green) was transiently transfected into knockdown cells to rescue the phenotype. Arrows: centrosome-localized exogenous TUFT1. Scale bar: 1 μm. (F) Quantification of SAS-6 and PLK4 foci numbers in (E) (n > 300 cells from three individual experiments). Data in (C), (D), and (F) are shown as the mean ± s.e.m. *P*-values are shown in the figure (one-way ANOVA).


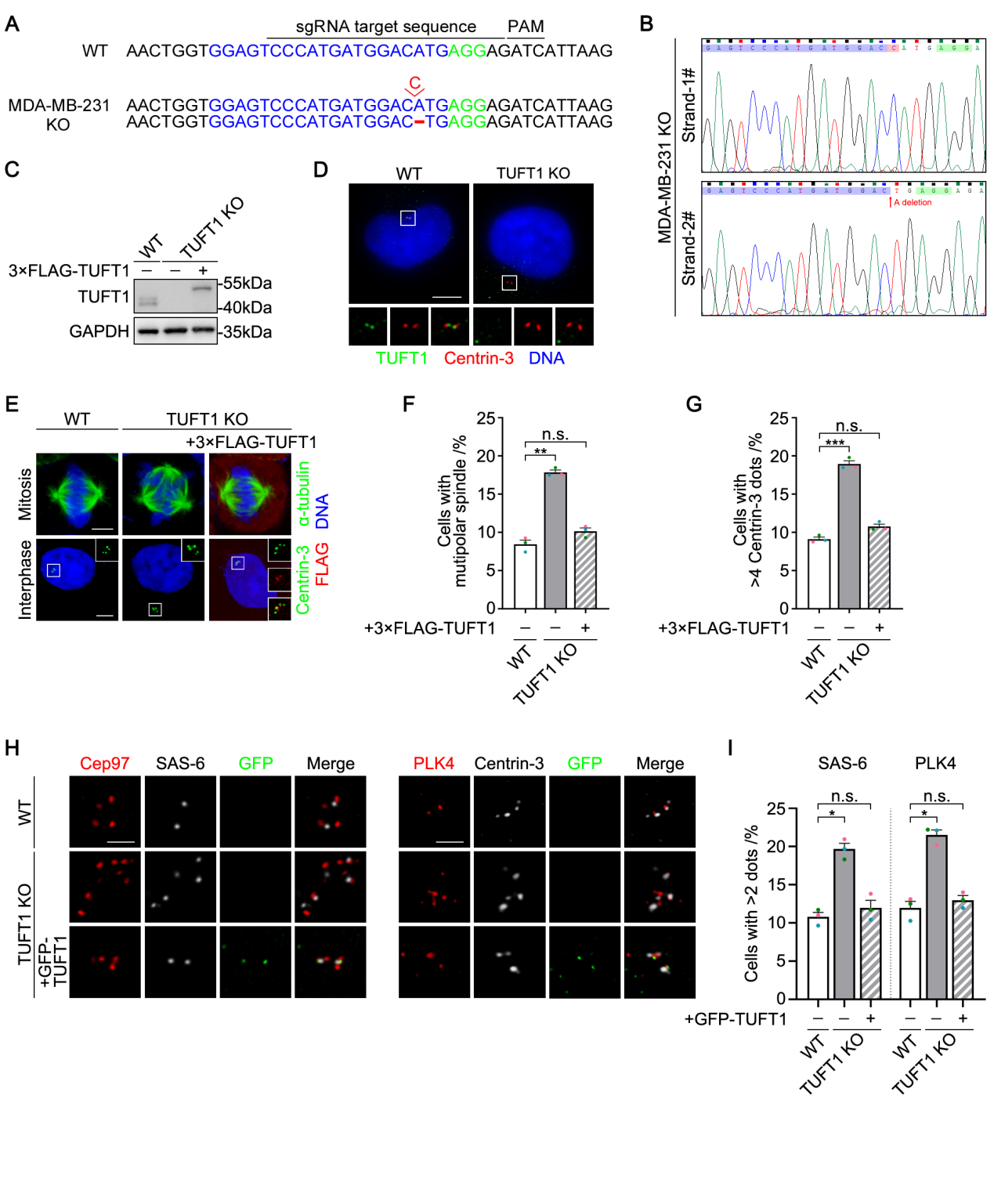


**Figure S6. TUFT1 knockout induces centrosome amplification and mitotic spindle multipolarity in MDA-MB-231 cells.** (A) Schematic of the TUFT1-knockout (KO) MDA-MB-231 cell line. The protospacer adjacent motif (PAM) sequence is highlighted in green, single guide RNA (sgRNA) target sequence is highlighted in blue, and mutated sequence is highlighted in red. (B) Genome sequence of the KO cell line around the targeted region. (C) Immunoblots of TUFT1 in wild-type (WT) and TUFT1-KO MDA-MB-231 cells. GAPDH served as a loading control. (D) Immunostaining of TUFT1 (green) and Centrin-3 (red) in WT and TUFT1-KO MDA-MB-231 cells. DNA was stained with DAPI (blue). Scale bar, 5 μm. (E) Immunostaining of α-tubulin (green, upper panel) or Centrin-3 (green, bottom panel) in WT or TUFT1-KO MDA-MB-231 cells. 3×FLAG-TUFT1 (red) was transiently transfected into KO cells to rescue the phenotype. DNA was stained with DAPI (blue). Scale bar, 5 μm. (F) Quantification of cells with multipolar spindle in (E) (n > 300 cells from three independent experiments). (G) Quantification of Centrin-3 foci number in (E) (n > 300 cells from three independent experiments). (H) Immunostaining of Cep97 (red, left panel), SAS-6 (white, left panel), PLK4 (red, right panel), and Centrin-3 (white, right panel) in WT and TUFT1-KO MDA-MB-231 cells. GFP-TUFT1 (green) was transiently transfected into KO cells to rescue the phenotype. Scale bar: 1 μm. (I) Quantification of SAS-6 and PLK4 foci numbers in (H) (n > 300 cells from three individual experiments). Data in (F), (G), and (I) are shown as the mean ± s.e.m. *P*-values are shown in the figure (one-way ANOVA).


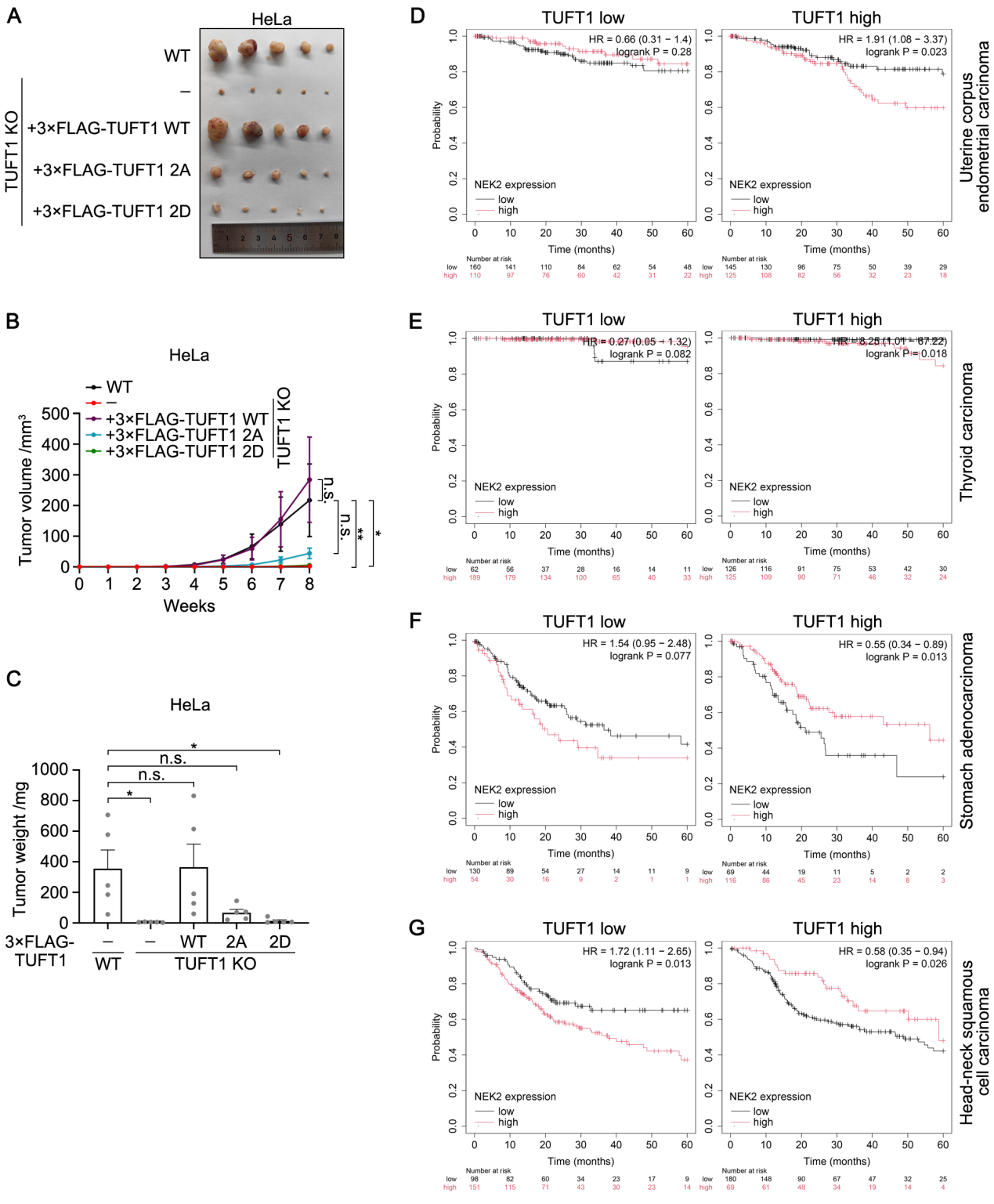


**Figure S7. Phosphorylation of TUFT1 regulates HeLa cell growth *in vivo* and correlates with patient outcomes in various cancers.** (A) WT and TUFT1-knockout (KO) HeLa cells that stably express 3×FLAG-TUFT1 (WT, 2A, or 2D) were subcutaneously injected into female nude mice to induce ectopic tumor formation (n = 5). Representative photograph of excised tumors is shown. (B) Quantification of the tumor volume in (A). (C) Quantification of the tumor weight in (A). (D-G) Correlation between overall survival and NEK2/TUFT1 expression in uterine corpus endometrial carcinoma (D), thyroid carcinoma (E), stomach adenocarcinoma (F), and head-neck squamous cell carcinoma (G). Cohorts of patients of indicated cancer types were collected from public databases and divided into two groups according to the median of TUFT1 expression. Data in (B) and (C) are shown as the mean ± s.e.m. *P*-values are shown in the figure (one-way ANOVA in (B) and (C); two-sided log-rank test in (D-G)).

**Supplementary table**

**Table S1.** **The clinical information of the TNBC cohort used for analysis.**


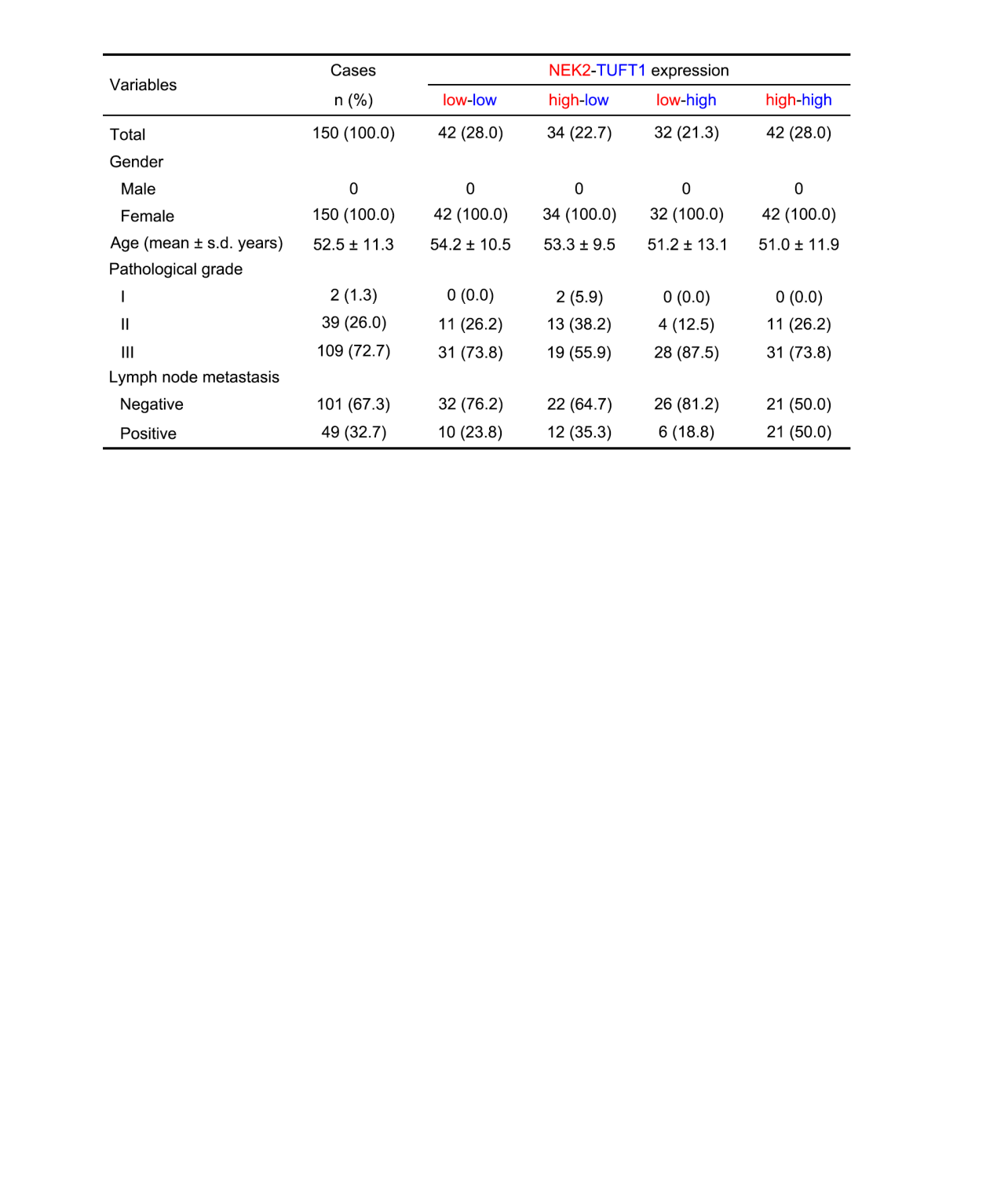

Supplement: Supplementary file 1 — Supplemental Figure and Table [file 41419_2025_8010_MOESM1_ESM.docx]
