# Supplementary figures and images for: TUFT1 regulates cancer progression by suppressing centrosome amplification and mitotic spindle multipolarity
Source: Cell Death Dis. 2025 Sep 29;16(1):673. doi: 10.1038/s41419-025-08010-3 (PMC12480466; doi:10.1038/s41419-025-08010-3)

Source data for western blot figures

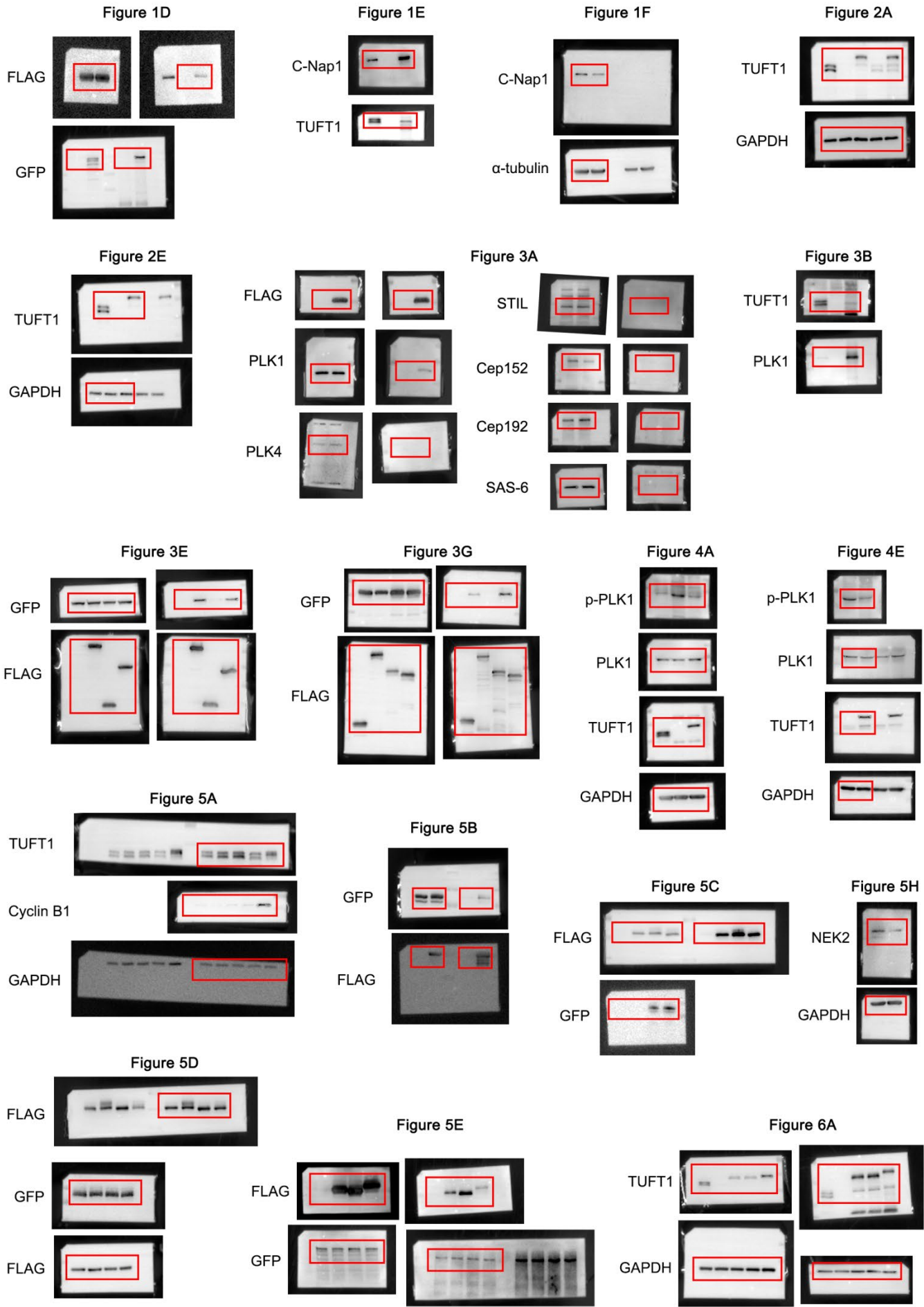

Source data for western blot figures

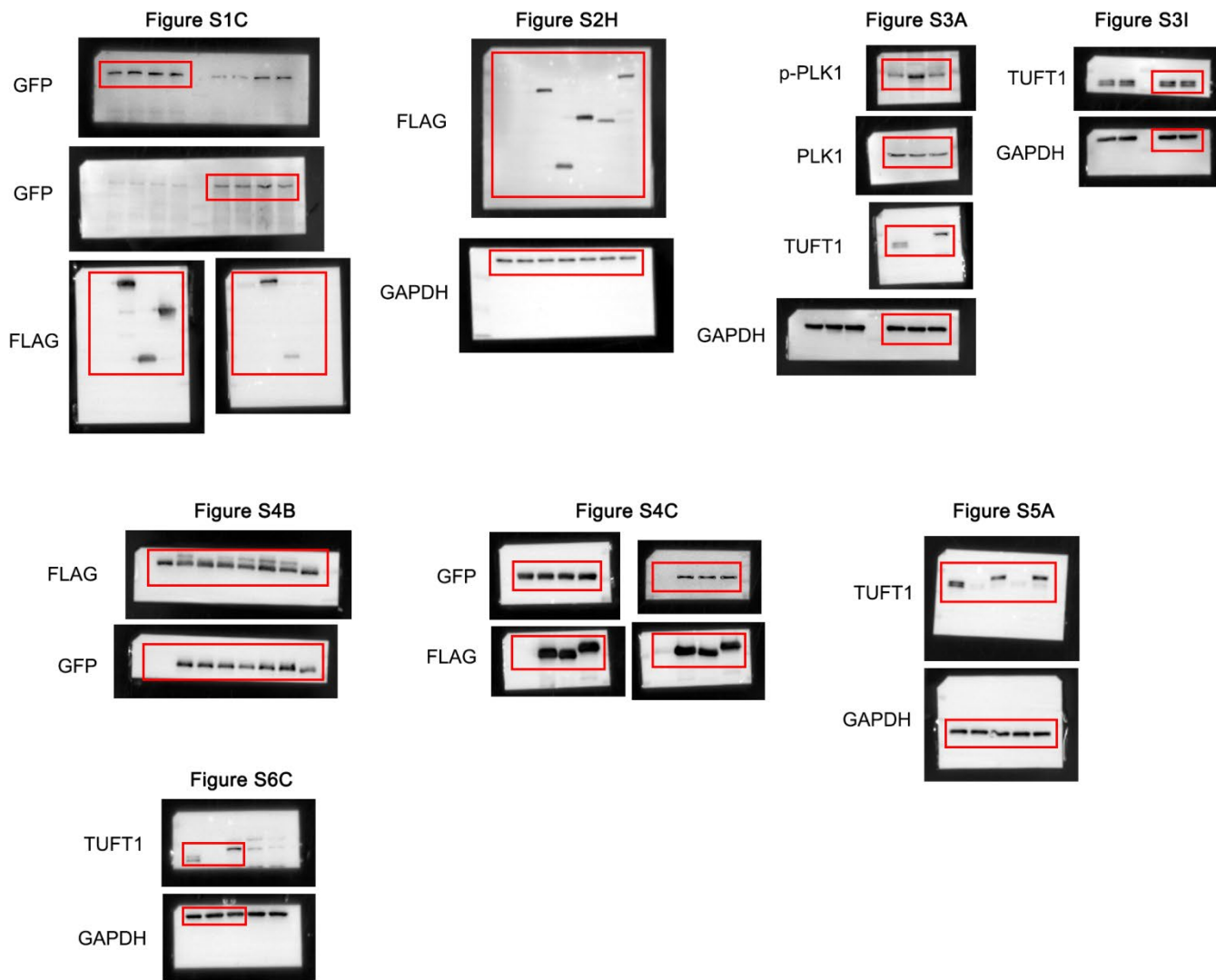

Supplement: Supplementary file 2 — Original Western blots [file 41419_2025_8010_MOESM2_ESM.pdf]
